# Clinical nurses’ knowledge, attitudes and practices regarding incontinence-associated dermatitis: A cross-sectional study from China

**DOI:** 10.1371/journal.pone.0337721

**Published:** 2025-12-09

**Authors:** Mufen Ye, Siyue Fan, Yanni Lin, Xiaoying Tong, Shuangling Kang, Xiaoxia Chen, Qiuni Cai, Huiling Zeng, Lijuan Chen

**Affiliations:** 1 Zhongshan Hospital of Xiamen University, School of Medicine, Xiamen University, Xiamen, China; 2 School of Nursing, Ningxia Medical University, Yinchuan, China; 3 Nursing College, Fujian University of Traditional Chinese Medicine, Fuzhou, China; Islamic Azad University, IRAN, ISLAMIC REPUBLIC OF

## Abstract

To explore the current status of knowledge, attitudes, and practices (KAP) regarding incontinence-associated dermatitis (IAD) among clinical nurses in China and identify factors influencing these three aspects, this study adopted a cross-sectional design and was conducted in a tertiary hospital in Fujian Province, China, with 1,153 nurses completing the questionnaire. Primary outcome measures included nurses’ IAD-related knowledge, attitude, and practice scores, with scoring rates of 70.6%, 90.9%, and 69.9% respectively; key low-scoring items in knowledge were “IAD care products” (2.04 ± 1.019) and “IAD assessment tools” (2.12 ± 0.923), while low-scoring practice items included “actively participating in IAD seminars” (60%) and “using IAD assessment tools” (66%). Demographic and professional information collected included gender, age, highest educational attainment, work experience, job position, job grade, department, wound/ostomy/incontinence (W/O/I) team membership, and participation in W/O/I care training. Results showed that clinical nurses had insufficient IAD knowledge (only 15.3% with high knowledge levels) and inadequate practices, but held positive attitudes (75.4% with positive attitudes). Notably, male nurses demonstrated more comprehensive IAD knowledge than female nurses (p < 0.05), though no significant gender difference was observed in practice (p = 0.883); this knowledge difference may be associated with male nurses’ more frequent assignment to IAD-high-incidence departments (e.g., ICUs) and higher participation in W/O/I training. Factors influencing nurses’ IAD-KAP included: 1) Common factors across KAP: job position (specialist nurses and head nurses performed better) and W/O/I team membership; 2) Dimension-specific factors: department affiliation (ICU nurses outperformed general ward nurses in knowledge and practice), participation in W/O/I training (improved knowledge and practice), gender (influencing knowledge only), and work experience (longer tenure correlated with more positive attitudes). Highest educational attainment was not a significant influencing factor, possibly due to inconsistent quality of part-time continuing education for nurses. In conclusion, participating nurses exhibited insufficient IAD knowledge and inadequate practices despite positive attitudes, presenting a notable “attitude-practice gap”. Nursing administrators should develop targeted training (e.g., scenario-based training on IAD assessment tools and care products) and encourage female nurses to rotate in high-incidence departments (e.g., ICUs), strengthen resource allocation (e.g., ensuring supply of IAD care products) and promote W/O/I team construction, and optimize continuing education quality to address knowledge disparities, thereby enhancing nurses’ understanding of IAD prevention and improving their related practices.

## Introduction

Incontinence-associated dermatitis (IAD) is an irritant dermatitis of the gluteal, sacrum-caudal, and perianal regions resulting from prolonged exposure to urine and feces [1]. It is characterized by diffuse erythema, usually with or without edema of the skin surface [2,3]. As one of the most prevalent forms of moisture-associated dermatitis, IAD has evolved into a global health concern, necessitating increased attention from healthcare professionals to focus on prevention and mitigation strategies [4]. The incidence of IAD varies significantly across different medical settings, with a total incidence ranging from 5.6% to 50.0% in various healthcare institutions and patient populations [5,6]: in acute care facilities, the prevalence is 19% [7]; in long-term care facilities, it is 30% [8]; and in Australian hospitals, 42% of patients with urinary and fecal incontinence (prevalence 24%) ultimately develop IAD [9]. In China, the prevalence of IAD ranges from 14.0% to 32.6%, a rate significantly higher than that in other countries [10]. Unfortunately, this data may be underestimated, the current lack of internationally standardized IAD assessment tools and unified data collection methods leads to incomplete recording of mild or early-stage IAD cases [11]. This high and potentially underreported incidence highlights the urgency of addressing IAD in the Chinese healthcare context.

IAD imposes substantial burdens on patients: it causes unnecessary pain, pruritus, sleep disturbances, and infections, directly impairing their psychological well-being and health-related quality of life [4]. More critically, delayed prevention and treatment of IAD may trigger pressure injuries (PI), which prolong patients’ hospital stays, increase their treatment costs, and add complexity to clinical nursing work [12,13]. In response to these harms, current research primarily focuses on developing therapeutic products (e.g., advanced skin protectants) and preventive strategies (e.g., scheduled skin assessment) [14]. However, these efforts have not yet yielded effective structured prevention and management protocols that can be widely implemented. In China, the situation is even more challenging: IAD preventive measures are often suboptimal, with a lack of systematic management and standardized care processes across most hospitals [15]. Only a limited number of institutions have incorporated IAD into routine nursing quality assessment.

The Knowledge-Attitude-Practice (KAP) model provides a framework to address this gap, it posits that healthcare professionals’ knowledge, attitudes, and practices are interconnected, with knowledge and attitudes directly shaping their practical behaviors [16]. As the primary providers of patient care, nurses maintain close and prolonged contact with patients, making them the core force in IAD prevention and treatment [17]. Thus, nurses’ KAP regarding IAD directly impacts the quality of care and patient outcomes. Knowledge refers to the mastery of a subject, which is acquired through clinical experience and research [11]. Existing studies suggest that due to insufficient knowledge, the majority of clinical nurses perform poorly in preventing IAD [10]. A thorough grasp of IAD-related knowledge by nurses is essential for the implementation of preventive care [11]. Beyond knowledge, nurses’ attitudes toward IAD also influence their care practices. Attitude is defined as the predisposition to face, resolve, and reflect upon issues in various contexts [18]; nurses with a positive attitude toward IAD prevention are more likely to adopt supportive practices, as a proactive mindset is closely linked to standardized preventive care [19]. Currently, global efforts are underway to investigate the KAP of clinical nurses regarding IAD, with studies from multiple countries providing insights into targeted training needs [20,21]. However, research in China remains limited, existing studies either use small samples or only explore partial KAP dimensions, failing to reflect the overall status of Chinese nurses.

Given the high incidence of IAD in China and its adverse effects on patients, this study aims to systematically explore the IAD-related KAP of Chinese clinical nurses. The findings will provide evidence-based data for nursing administrators, supporting the development of targeted training programs. Ultimately, this will enhance Chinese nurses’ KAP of IAD and improve the quality of IAD prevention in clinical settings.

## Materials and methods

### Study design and setting

This cross-sectional study was conducted among 1,153 clinical nurses working at a tertiary general hospital in Fujian, China. The recruitment period for participants lasted from June 12, 2024 to July 12, 2024, during which eligible nurses were invited to participate in the questionnaire survey. All study procedures strictly adhered to the ethical principles outlined in the Declaration of Helsinki (World Medical Association, 2022).

The current study is a sub-project of the larger research initiative titled “Construction of a risk prediction model for incontinence-associated dermatitis in elderly patients based on machine learning,” which has obtained formal ethical approval from the Institutional Review Board (IRB) of Hospital (Approval No. 2024−055). Per the IRB’s official guidelines, the ethical approval for the larger project explicitly extends to all sub-projects under its framework, including the present study focusing on “Clinical Nurses’ Knowledge, Attitudes and Practices Regarding Incontinence-Associated Dermatitis.” This coverage is justified by the fact that the current study shares key methodological and ethical consistency with the larger project: identical research population (clinical nurses), consistent data collection method (anonymous questionnaire surveys), and adherence to the same ethical safeguards (e.g., voluntary participation, electronic informed consent, de-identification of data for privacy protection). Thus, no separate ethical review or approval was required for the current study.

Prior to questionnaire completion, all participants provided informed consent in electronic form (consistent with the larger project’s ethical approval requirements). Specifically, participants first accessed a detailed electronic study information sheet via the questionnaire platform, which outlined the study’s purpose, data collection procedures, potential risks/benefits, and the right to withdraw at any time without penalty. Only after confirming understanding and clicking the “I agree to participate” button, with the consent action automatically recorded by the platform (including a time stamp and the participant’s device IP address for traceability), were participants allowed to proceed with the questionnaire. All participants were adults (≥18 years old), and no minors were included in the study, so consent from parents or guardians was not required. The need for informed consent was not waived by the IRB; electronic consent was formally approved as a valid consent method in the larger project’s ethical approval document.

### Participants

The entire nursing staff of a tertiary general hospital in Fujian, China, with more than 2,000 beds and functioning as a regional medical center, comprised the study’s target population. A convenient sampling method was used to select clinical nurses who volunteered to participate in the study.

To minimize selection bias, the research team implemented the following sampling procedures: (1) Before the survey launch, the hospital’s nursing department provided a list of all eligible nurses (including their affiliated departments and job titles) to ensure the sampling frame covered all clinical departments (e.g., internal medicine, surgery, geriatrics, ICU, and outpatient care) without exclusion; (2) The questionnaire QR code was shared simultaneously in all department-specific nurse WeChat groups on June 12, 2024, to avoid delayed access by nurses from specific departments; (3) Daily reminders (no more than 1 reminder per day) were sent by department head nurses between June 12 and July 12, 2024, reminders were standardized (same text content) to prevent over-recruitment of nurses from departments with more frequent reminders.

The inclusion and exclusion criteria for participants were as follows:

Inclusion criteria included: (1) being a registered nurse (valid registration certificate verified by the hospital nursing department); (2) having at least six months of clinical work experience (calculated from the date of formal employment); (3) volunteering to participate in the study (confirmed via electronic informed consent).

Exclusion criteria included: (1) nurses from other hospitals who come to our hospital for further education (duration <6 months); (2) providing insufficient information in the questionnaire, including either missing key data on essential study variables (e.g., job position, membership in the wound/ostomy/incontinence [W/O/I] team) or incomplete responses to more than 15% of the total questionnaire items, which would compromise the reliability of the K-A-P assessment.

### Instruments

#### Demographic and professional information.

A self-report questionnaire was used to gather data on the general (gender, age, education level, work experience, position, job grade, and department) and work-related characteristics (attendance at W/O/I training courses and membership in the W/O/I team) of the participants.

**Nursing staff IAD knowledge, attitude, and practice questionnaire:** Utilizing the IAD Knowledge, Attitude, and Practice Questionnaire developed by Chinese scholar Kong Jie, this study assesses the KAP levels of clinical nurses regarding IAD [22]. The full Chinese original version and English translated version of the questionnaire are available in S1 File, ensuring consistency with the item descriptions and scoring rules outlined below. No modifications were made to the questionnaire’s core items (knowledge, attitude, practice dimensions) to ensure consistency with the original validation standards.

The development of this questionnaire followed a rigorous process based on the KAP theory: first, a systematic literature review (covering international IAD care guidelines and published scales) and semi-structured interviews with 10 clinical nurses (including 3 members of the W/O/I team) were conducted to initially generate 27 items across the three core dimensions of knowledge, attitude, and practice; second, two rounds of Delphi consultations were carried out with 5 experts (specializing in nursing management, clinical nursing, and W/O/I care, with an average authority coefficient of 0.87) to optimize the items, 1 item with an agreement rate <75% was deleted, and 2 items with ambiguous expressions were revised, ultimately confirming 26 items; third, a pre-survey involving 130 clinical nurses was implemented to verify construct validity: the KMO test value was 0.861, Bartlett’s spherical test *χ*² = 2675.648 (*p* < 0.001), and 3 common factors were extracted via maximum variance orthogonal rotation, with a total cumulative variance contribution rate of 59.274%, which was consistent with the initial three-dimensional framework.

The questionnaire encompasses three dimensions: knowledge, attitude, and practice, comprising a total of 26 items with a scoring range of 26–104 points. The knowledge dimension consists of 13 items, and the scoring design is tailored to the nature of IAD knowledge content (factual vs. conceptual familiarity): Factual knowledge items (Items 2–4, 8–13): These items assess objective, operational knowledge (e.g., “Which factors are associated with the occurrence of IAD?” “Which body fluids cause the most skin damage?”). For these, a “correctness-based scoring” approach is used: each item scores 4 points for a fully correct answer. If an answer is incorrect or partially correct, 1 point is deducted for each wrong or missing option, with a minimum score of 0. This design directly reflects the mastery of clinical facts, ensuring the objectivity of knowledge measurement. Conceptual familiarity items (Items 1, 5–7): These items evaluate familiarity with conceptual knowledge (e.g., “How well do you understand the definition of IAD?” “Are you familiar with IAD grading?”). A 4-point Likert scale is adopted, ranging from “familiar” (4 points) to “unfamiliar” (1 point). The rationale is that such conceptual knowledge involves “degrees of familiarity” rather than absolute “right/wrong.” To minimize subjectivity, clear criteria for each Likert level were defined in the pre-survey (e.g., “familiar” means being able to accurately state the core definition; “somewhat familiar” means knowing key keywords but not the full definition), and investigators were trained to guide participants during scoring. The attitude dimension, which comprises 7 items, utilizes a 4-point Likert scale, from “strongly agree” (4 points) to “disagree” (1 point). The practice dimension, consisting of 6 items, adopts a 4-point Likert scale, extending from “frequently” (4 points) to “never” (1 point).

Three dimensions of Cronbach’s *α* yielded values of 0.889, 0.879, and 0.884, with a total Cronbach’s *α* of 0.889, indicating moderate to high internal consistency reliability; the external reliability (test-retest reliability, measured among 30 nurses with a 15-day interval) of the questionnaire was evaluated at 0.837, while the mean CVI fluctuated within the range of 0.9 to 1 and the content validity (via expert consultation) was 0.969, suggesting satisfactory reliability and validity and appropriateness for a KAP survey among clinical nursing personnel.

The scoring rate was calculated as follows: scoring rate equals the average score divided by the perfect score, multiplied by 100 percent. Thresholds for level classification based on scoring rate were defined as follows: a scoring rate higher than 80 percent was defined as good (corresponding to a positive level, e.g., “good knowledge mastery” for the knowledge dimension, “positive attitude” for the attitude dimension, “standard practice” for the practice dimension), a scoring rate ranging from 60 percent to 80 percent was defined as medium (corresponding to a neutral level, e.g., “moderate knowledge mastery,” “neutral attitude,” “basically standardized practice”), and a scoring rate lower than 60 percent was defined as poor (corresponding to a negative level, e.g., “poor knowledge mastery,” “negative attitude,” “non-standard practice”) for each dimension and the total score.

#### Data collection.

**Survey platform and timeline:** The questionnaire was distributed online via a professional survey platform (http://www.wjx.cn; official certification number: GS11010520220001, verified for data security compliance by the hospital’s information technology department). The survey period was from June 12, 2024, 09:00 (Beijing Time) to July 12, 2024, 23:59 (Beijing Time), a 30-day window to ensure sufficient time for nurses working different shifts (day, night, and rotating shifts) to participate.

A unique, non-reusable QR code linking to the questionnaire was generated for this study (QR code validity period synchronized with the survey timeline). The QR code was shared through hospital-official nurse WeChat groups (administered by the nursing department, not private groups) to ensure only eligible nurses from the target hospital could access the questionnaire, external access was restricted by the platform’s IP filtering function (only IP addresses within the hospital’s intranet and the nurses’ registered mobile phone IPs were allowed).

***Department head training and promotion support:*** Prior to the survey launch (June 10, 2024), the research team organized a 90-minute offline training session for the head nurses of all departments. During the session, detailed information about the study was provided, including the research purpose, participant eligibility criteria, instructions for accessing the questionnaire, and key precautions (e.g., ensuring anonymous response and data privacy protection).The training included: (1) A detailed presentation of the study protocol (approved by the IRB, No. 2024−055), including the rationale for convenient sampling and measures to avoid bias; (2) A hands-on demonstration of accessing the questionnaire via the QR code, with troubleshooting guidance for common issues (e.g., QR code scanning failure, page loading errors); (3) Provision of a standardized reminder template (e.g., “Dear colleagues, the IAD KAP survey is ongoing until July 12. If you have time, please complete it voluntarily via the QR code shared earlier. Thank you for your support!”) to ensure consistent communication across departments.

After the training, each department head nurse was asked to assist in disseminating the survey information to nurses within their department (e.g., re-sharing the questionnaire QR code and reminding nurses of the survey timeline) and to answer basic questions about the survey process (e.g., how to access the questionnaire if a link failed). Notably, department heads were explicitly instructed not to urge or require nurses to complete the questionnaire, to ensure participation remained voluntary. After the training, each head nurse signed a “Survey Promotion Commitment Form” to confirm they would not coerce or incentivize participation (e.g., no association with job performance evaluations). The research team conducted random spot checks (5 departments per week) to verify compliance.

**Informed consent and questionnaire completion guidelines:** Before accessing the questionnaire, all potential participants were presented with a detailed electronic informed consent form, which outlined the study background, purpose, data usage (for research only), privacy protection measures (e.g., anonymous data storage and de-identification), and the right to withdraw from the survey at any time without consequences. Only after participants clicked “I agree” to the consent form could they proceed to complete the questionnaire anonymously using electronic devices such as laptops or mobile phones.

Additionally, the introduction section of the questionnaire re-emphasized the voluntary nature of participation, expressed gratitude for their involvement, and included a statement urging participants to complete the questionnaire independently based on their actual clinical knowledge, attitudes, and practices, explicitly requesting that they avoid using artificial intelligence tools, reference materials, or consulting with others during completion, as this could lead to biased data that does not reflect their true KAP levels. For any questions related to the questionnaire content during completion, participants could directly contact the research team for clarification.

**Data quality control measures:** Regarding the potential risk of questionnaire completion using artificial intelligence (AI) or external resources, the research team took targeted mitigation measures:

First, the questionnaire’s knowledge dimension included scenario-based items closely tied to clinical practice (e.g., “Which skin cleaning solution is most appropriate for IAD patients in your daily care?”), such items require familiarity with real-world nursing operations, making it difficult for generic AI tools or external references to provide answers consistent with individual clinical experience.

Second, the survey platform was set to: (1) limit each IP address and registered mobile phone number to one questionnaire submission, preventing repeated attempts to “optimize” answers; (2) record the completion time for each item (not just total time) to identify abnormal patterns (e.g., < 10 seconds per item for complex scenario questions).

Third, during data preprocessing, we applied the following exclusion criteria for invalid responses: (1) total completion time <3 minutes, far below the average 8–10 minutes required for thoughtful completion; (2) identical answers to all Likert-scale items (e.g., all “strongly agree” in the attitude dimension); (3) contradictory responses (e.g., selecting “never participated in W/O/I training” but answering “I apply barrier cream based on training content”);

These measures collectively reduce the impact of non-independent completion on data validity, ensuring the survey results accurately reflect clinical nurses’ true IAD-KAP levels.

### Data analysis

**Analysis tool:** For data analysis, IBM’s Statistical Package for Social Science (SPSS; Version 27.0) was utilized as the primary statistical software. All statistical tests were planned to two-tailed, and the process of variable selection, confounding control, and model validation was documented in detail to ensure reproducibility. Intermediate results (e.g., univariate analysis outcomes, residual statistics) would be summarized in dedicated tables in the Results section.

**Descriptive statistical analysis:** Descriptive statistics were planned to characterize the demographic and professional attributes of participants and summarize the distribution of IAD-KAP scores. The analytical strategy was pre-defined as follows:

(1) Categorical variables (e.g., gender, work department, membership in the W/O/I team): To be reported as frequency (n) and percentage (%), with categories aligned to the actual clinical structure of the study hospital (e.g., work department grouped into surgery, internal medicine, emergency room, and ICU).
(2) Continuous variables (e.g., age, total KAP score): To be reported as mean ± standard deviation. Age was pre-stratified into 4 groups (20–30, 31–40, 41–50, ≥ 51 years) and work experience into 5 groups (<5, 6–10, 11–15, 16–20, ≥ 20 years), consistent with the grouping plan for subsequent univariate analysis.
(3) KAP dimension scores: To be calculated per the scoring rules described in “Nursing staff IAD knowledge, attitude, and practice questionnaire” Section. Scoring rates (average score/perfect score × 100%) were pre-specified to classify proficiency levels: high (>80%), moderate (60%–80%), and low (<60%).

**Univariate analysis:** Univariate analysis was designed to explore differences in nurses’ IAD-KAP levels (knowledge, attitude, practice, total score) across groups of demographic and professional characteristics. The analytical framework was pre-defined based on variable type and group number:

(1) Two-group comparisons (e.g., gender, W/O/I team membership, W/O/I care training participation): Independent samples t-tests were designated for normally distributed KAP scores (normality to be verified via the Shapiro-Wilk test, with a pre-specified threshold of p > 0.05). Two-sided Mann-Whitney U tests were reserved for non-normally distributed data (to be used if the Shapiro-Wilk test indicated p ≤ 0.05).
(2) Multiple-group comparisons (e.g., age, work experience, job grade, work department): One-way analysis of variance (ANOVA) was planned for normally distributed data. Post-hoc pairwise comparisons were set to apply the Bonferroni correction to control Type I error, with the adjusted significance level calculated as *α*’ = 0.05/n (n = number of pairwise comparisons; e.g., 4 age groups would require 6 comparisons, leading to *α*’ = 0.05/6 ≈ 0.008). Kruskal-Wallis H tests were reserved for non-normally distributed data (to be used if ANOVA assumptions were violated).
(3) Candidate variable screening for multivariate analysis: Variables with a pre-specified threshold of *p* < 0.2 in univariate analysis were planned to be retained as candidate independent variables for subsequent regression models. This liberal threshold was chosen to avoid missing potential confounders. Variables with *p* ≥ 0.2 were designed to be excluded from further multivariate analysis.

Candidate variable screening for multivariate analysis will be guided by both pre-specified statistical thresholds and the KAP model, ensuring the selection process is not purely statistically driven but aligned with the study’s theoretical basis:

Theoretical basis for variable inclusion (rooted in KAP model): Variables reflecting “knowledge accumulation capacity”: Age, work experience, and job grade are pre-selected as candidate variables. Per KAP model, nurses’ knowledge of IAD is primarily acquired through prolonged clinical practice and career development, longer work experience and higher job grades typically correspond to more accumulated IAD-related knowledge, which further shapes attitudes and practices. Variables reflecting “access to knowledge/practice resources”: W/O/I team membership and W/O/I care training participation are prioritized. The Introduction highlights that “insufficient knowledge leads to poor IAD prevention practices” and “targeted training improves KAP levels”, W/O/I team membership provides continuous exposure to specialized IAD care scenarios, while training directly delivers evidence-based knowledge, both of which are key drivers of KAP improvement per KAP model. Variables reflecting “practice context”: Work department and work position are included. Clinical context (e.g., high-IAD-prevalence departments like internal medicine/surgery) affects nurses’ frequency of IAD care practice, while work position (e.g., head nurse vs. staff nurse) influences access to care protocols and implementation of standardized practices, both align with KAP model’s emphasis on “context shaping practice.”

Statistical threshold for variable retention: Consistent with pre-planned criteria to avoid missing theoretically relevant variables, only variables with a *p* < 0.2 in univariate analysis will be retained as candidate independent variables for multivariate regression. Variables with *p* ≥ 0.2 (indicating weak association with KAP dimensions and no theoretical justification for retention) will be excluded from further analysis.

This dual (theoretical and statistical) screening approach ensures candidate variables are both clinically meaningful (per KAP model) and statistically relevant, laying a rigorous foundation for subsequent multivariate regression analysis.”

(4) Potential confounders were pre-defined to be identified via two criteria: (1) Clinical relevance: Variables reported in peer-reviewed studies on IAD-KAP as factors associated with nurses’ professional competence; (2) Statistical association: Variables expected to correlate with both candidate independent variables (e.g., work department) and dependent variables (KAP scores), to be assessed via preliminary Pearson correlation analysis (with a pre-specified threshold of *|r|* > 0.2 or *p* < 0.2). The final list of confounders (to be confirmed via the above criteria) was planned to include work experience (continuous), job grade (ordinal), W/O/I team membership (dichotomous), and W/O/I training participation (dichotomous), all of which were designed to be retained in multivariate models.

**Correlational analysis:** Pearson product-moment correlation analysis was pre-specified to explore linear associations between the three IAD-KAP dimensions (knowledge, attitude, practice). The analytical plan included: (1) Inclusion of all eligible participants (with complete KAP score data). (2) Reporting of correlation coefficients *|r|* and two-tailed *p*, with statistical significance interpreted as *p* < 0.05 (pre-specified threshold). (3) Classification of correlation strength using conventional thresholds: weak (*|r|* = 0.1–0.3), moderate (*|r|* = 0.3–0.5), and strong (*|r|* > 0.5), to ensure consistent interpretation of results.

**Multivariate stepwise multiple linear regression analysis: *Rationale for selecting stepwise regression:*** Stepwise multiple linear regression was pre-selected to identify factors influencing IAD-KAP scores, based on three pre-specified rationales: (1) Candidate variables (to be listed in an independent variable assignment table in the Results section) are anticipated to include highly correlated pairs (e.g., work experience and job grade). Stepwise regression (pre-set entry criterion *p* < 0.05, removal criterion *p* > 0.10) is planned to sequentially include only variables with significant predictive power, avoiding overfitting caused by manual inclusion of collinear variables. Multicollinearity will be further verified via the Variance Inflation Factor (VIF) in the Results section, with a pre-specified acceptable threshold of *VIF* < 5. (2) Alignment with study objectives: The study’s primary goal is to identify actionable clinical factors (e.g., training programs, team membership) for IAD care improvement, rather than maximizing model explanatory power. Stepwise regression is designed to prioritize variables by maximizing *R²* change, ensuring the final model is concise and interpretable for clinical application, even for models with low adjusted *R²* (e.g., attitude dimension), where the focus remains on “identifying meaningful predictors” rather than “explaining variance, and potential unmeasured confounders (e.g., organizational support policies) will be discussed to address model limitations”. (3) Theoretical-methodological consistency: Stepwise selection aligns with KAP model: it will retain variables that fit theoretical constructs (e.g., W/O/I training as a “knowledge acquisition facilitator” factor) and exclude those without theoretical relevance (e.g., non-modifiable demographic variables with no intervention value), ensuring the model’s clinical utility.

***Implementation details of regression analysis*:** Four regression models were designed (with dependent variables as knowledge, attitude, practice, and total KAP scores, respectively), with pre-specified parameters as follows: (1) Variable preprocessing (to be detailed in a dedicated independent variable assignment table in the Results section): Dichotomous variables: Gender, W/O/I team membership, and W/O/I training participation were planned to be coded as 0 (reference group) and 1 (exposed group) during analysis (e.g., “Male” = 0, “Female” = 1; “No training” = 0, “Training” = 1), to clarify the interpretation of regression coefficients. Ordinal variables: Job grade (N0–N4) and work position (staff nurse, education nurse, head nurse, wound care nurse) were designed to be treated as continuous variables only after verifying proportional odds via ordinal logistic regression (with a pre-specified threshold of *p* > 0.05 for acceptability). Categorical variables with >2 groups: Age (4 groups), work experience (5 groups), and work department (4 groups) were planned to be retained as ordinal variables for simplicity in coefficient interpretation. Continuous variables: Age and work experience were pre-specified to be centered (by subtracting the mean) to reduce multicollinearity with potential interaction terms and improve the interpretability of regression coefficients (e.g., “change in KAP score per 1-year increase relative to the mean age/work experience”). (2) Model input variables: Independent variables: Candidate variables with *p* < 0.2 in univariate analysis, plus work experience (designated for forced entry as a confounder, based on clinical relevance). Dependent variables: Raw KAP scores (not standardized), to preserve clinical interpretability of coefficient magnitudes (e.g., “change in actual knowledge score” rather than standardized units). (3) Statistical control: Variable entry (*p* < 0.05) and removal (*p* > 0.10) criteria were pre-set to reduce the risk of false-positive results in multiple variable tests. The overall significance of each final model was planned to be tested via F-test. Multicollinearity: The *VIF* was pre-specified to be calculated for all included variables, with a threshold of *VIF* < 5 to confirm the absence of severe multicollinearity. To verify the robustness of stepwise regression results, a full model analysis (including all pre-selected candidate variables) will be conducted as a supplementary step. Key comparison indicators between stepwise models and full models will include: a. adjusted *R²* (to assess explanatory power gain); b. *VIF* (to evaluate multicollinearity differences); c. number of significant variables (to confirm whether stepwise selection excludes redundant predictors).

***Validation of linear regression assumptions*:** Core linear regression assumptions were planned to be verified via residual-based analyses, with pre-defined procedures and thresholds:

Independence of observations: To be verified via two checks: (1) Confirmation of no repeated measurements (each participant contributing one questionnaire); (2) Durbin-Watson test (with a pre-specified acceptable range of 1.5–2.5), to rule out autocorrelation.

Homoscedasticity (constant residual variance): To be assessed via two methods: (1) Scatter plots of standardized residuals (ZRESID) vs. standardized predicted values (ZPRED), pre-specified to confirm no “funnel-shaped” or “horn-shaped” trends (which would indicate heteroscedasticity); (2) Breusch-Pagan test (with a pre-specified threshold of *p* > 0.05), to statistically confirm homoscedasticity.

Residual normality: To be verified via: (1) Quantile-quantile (Q-Q) plots, pre-specified to confirm data points approximately align with the diagonal line of the theoretical normal distribution; (2) Assessment of standardized residual ranges (with a pre-specified threshold of ±4), slight deviations at the tails were deemed acceptable for large sample sizes (per the central limit theorem).

No extreme outliers: Residuals were planned to be screened using standardized residual values, with a pre-specified exclusion threshold of *|ZRESID|* > 4. Any outliers exceeding this threshold would be evaluated for potential data entry errors before final model fitting.

**Statistical significance criterion:** Statistical significance was pre-defined as two-tailed *p* < 0.05, with adjustments for multiple comparisons where applicable: Univariate post-hoc tests: Bonferroni correction (*α’* = 0.05/n, as detailed in “Univariate analysis” Section), to control Type I error across pairwise comparisons. Multivariate regression: 95% confidence intervals (CIs) for regression coefficients were planned to be reported, with *CIs* excluding 0 interpreted as statistically significant (consistent with the *p* < 0.05 threshold).

#### Patient and public involvement.

The current study only included clinical nurses as participants, so there were no patients involved.

## Results

### Nurse characteristics

A total of 1153 nurses from a tertiary public hospital in Fujian Province were enrolled, with demographic and professional profiles consistent with those of clinical nursing teams in Chinese tertiary healthcare institutions (Table 1), this alignment ensures the generalizability of study findings to similar clinical settings where IAD care is primarily delivered.

**Table 1:** Demographic and professional information of the participants (N = 1153).

Characteristics	Total, n (%)	
**Gender**	Female	1109 (96.2)
	Male	44 (3.8)
**Age (years)**	20-30	407 (35.3)
	31-40	526 (45.6)
	41-50	171 (14.8)
	≥51	49 (4.2)
**Work experiences (year)**	<5	249 (21.6)
	6-10	297 (25.8)
	11-15	305 (26.5)
	16-20	136 (11.8)
	≥20	166 (14.4)
**Job grade**	N0^a^	118 (10.2)
	N1^b^	208 (18.0)
	N2^c^	208 (18.0)
	N3^d^	544 (47.2)
	N4^e^	75 (6.5)
**Highest level of education**	Associate degree	201 (17.4)
	Undergraduate	934 (81.0)
	Master and above	18 (1.6)
**Nurses work position**	Staff nurse (clinical)	1063 (92.2)
	Education	45 (3.9)
	Head nurse (management)	41 (3.6)
	Wound care nurse	4 (0.3)
**Work department**	Department of surgery	453 (39.3)
	Department of internal medicine	564 (48.9)
	Emergency room	48 (4.2)
	ICU	88 (7.6)
**Are you a member of a wound/ostomy/incontinence team?**	Yes	70 (6.1)
	No	1083 (93.9)
**Have you attended any wound/ostomy/incontinence care training?**	No	463 (40.2)
	Yes	690 (59.8)

^a^A newly graduated nurse who has joined the work.

^b^Nurses who have worked in clinical practice for 1 year or more, obtained college education or above and obtained the qualification certificate of nurses. It is also necessary to meet the necessary requirements.

^c^Nurses who have worked in clinical practice for 3 years or more and have the title of junior nurse or above. It is also necessary to meet the necessary requirements.

^d^Nurses who have been engaged in clinical work for 5 years or more and have the title of charge nurse or above. Necessary conditions must also be met. N4: Nurses with the title of co-chief nurse.

Gender distribution showed 96.2% (1109/1153) of participants were female, which reflects the typical gender composition of China’s nursing workforce. Clinically, this is meaningful because female nurses predominantly undertake direct bedside care tasks, including routine skin assessments, incontinence management, and patient hygiene care, making their IAD-KAP levels directly tied to institutional performance in IAD prevention and patient skin safety.

By age, 80.9% of nurses fell within the 20–40 years range (35.3% aged 20–30 years, 45.6% aged 31–40 years), a cohort recognized as the “core clinical workforce” in Chinese hospital settings. This group typically bears approximately 70% of daily patient care workload, and their proficiency in IAD care is critical to reducing hospital-acquired IAD: they are the primary caregivers for high-risk populations (e.g., elderly bedridden patients, post-operative individuals with limited mobility) who are most susceptible to IAD.

Work experience and job grade further highlighted a sample of experienced clinicians: 52.3% had 6–15 years of clinical tenure (25.8% with 6–10 years, 26.5% with 11–15 years), and 47.2% (544/1153) held the N3 grade, China’s mid-to-senior clinical nursing rank, which denotes independent clinical decision-making capacity. Nurses in this experience and grade bracket often serve as mentors to junior staff; their IAD-KAP levels can therefore indirectly influence the care practices of the broader nursing team, emphasizing the value of targeting this group for IAD education and training initiatives.

Department affiliation underscored the study’s direct relevance to clinical IAD care needs: 88.2% of nurses worked in internal medicine (48.9%) or surgery (39.3%), two specialties with inherently high IAD prevalence. Internal medicine typically cares for elderly patients with higher rates of urinary/fecal incontinence, while surgery manages post-operative patients with limited mobility and indwelling medical devices (e.g., urinary catheters, drainage tubes), both factors increase patient exposure to prolonged skin moisture, a key trigger for IAD. The overrepresentation of nurses from these departments ensures study findings address the populations most directly involved in IAD care.

Notably, only 6.1% (70/1153) of participants were members of the W/O/I team, and 59.8% (690/1153) had attended W/O/I care training. This gap in specialized training coverage provides important context for interpreting subsequent KAP results: limited access to W/O/I-specific education (which focuses on evidence-based IAD assessment, staging, and intervention) may contribute to variability in IAD knowledge and practice across the sample. Addressing this training gap could therefore be a priority for improving overall IAD care quality in the study setting.

### Nurses’ KAP levels regarding IAD

The IAD-KAP scores of 1153 nurses revealed dimension-specific strengths and critical gaps, with direct implications for optimizing clinical IAD care (Table 2). Key findings and their clinical relevance are summarized below:

**Table 2:** The two highest and lowest scoring items for nurses’ knowledge, attitudes, and practices related to IAD.

Dimension	The item with the highest score	Score ($\overset{―}{X}\;\pm \;𝐬$)	The item with the lowest score	Score (<<Eqn2>>)
**Knowledge dimension**	What do you think of the key points of prevention and care for IAD?	3.93 ± 0.361	What products do you think can be used in IAD care?	2.04 ± 1.019
	Which of the following steps do you think are important to prevent IAD?	3.93 ± 0.454	Are you aware of any IAD Assessment Tool?	2.12 ± 0.923
**Attitude dimension**	Do you think it is important to strengthen the caregiving skills of family members/nursing assistants in the prevention and treatment of IAD?	3.71 ± 0.521	Do you think it is important for hospitals to conduct training on IAD knowledge?	3.52 ± 0.627
	Do you think IAD prevention is more important than treatment?	3.69 ± 0.553	Do you think it is important to attend training to improve nurses’ attitudes towards IAD prevention?	3.56 ± 0.604
**Practice dimension**	Have you adopted skin protection tools?	3.25 ± 0.681	Have you actively attended any academic lectures or training on IAD?	2.41 ± 0.750
	Have you conducted targeted education for family members/nursing assistants about IAD?	2.88 ± 0.723	Have you used IAD assessment tools?	2.64 ± 0.755

#### Knowledge dimension.

Scores ranged from 13 to 52 (mean: 36.76; scoring rate: 70.6%), with 15.3% (176/1153) of nurses demonstrating high knowledge, 72.2% (832/1153) moderate knowledge, and 12.5% (145/1153) low knowledge. The 70.6% scoring rate indicates suboptimal mastery of IAD-related knowledge, which poses tangible clinical risks: nurses with low knowledge may misclassify IAD stages (e.g., confusing mild IAD with PI) or select inappropriate skin care products (e.g., irritant cleansers that disrupt the skin’s protective barrier), directly increasing the likelihood of patient skin damage worsening or secondary infections. The high proportion of nurses with moderate knowledge (72.2%) also signals an opportunity, targeted knowledge reinforcement (e.g., focusing on IAD pathophysiology and staging criteria) could rapidly elevate overall proficiency to meet clinical safety needs.

#### Attitude dimension.

Scores ranged from 7 to 28 (mean: 25.45; scoring rate: 90.9%), with 75.4% (869/1153) of nurses showing high attitudes toward IAD care, 21.7% (250/1153) moderate attitudes, and only 2.9% (34/1153) low attitudes. This high attitude scoring rate reflects strong recognition among nurses that IAD is a preventable patient harm, an important foundation for subsequent practice improvement. However, it also highlights a potential “attitude-practice disconnect”: positive intentions toward IAD care do not guarantee consistent implementation, which may stem from practical barriers (e.g., high nurse-to-patient ratios limiting time for skin assessments, or lack of standardized IAD care protocols in daily workflows).

#### Practice dimension.

Practice dimension scores ranged from 6 to 24 (mean: 16.77; scoring rate: 69.9%), the lowest among all dimensions, with 16.3% (188/1153) of nurses achieving high practice levels, 60.2% (694/1153) moderate levels, and 23.5% (271/1153) low levels. This low practice proficiency is clinically concerning, as suboptimal IAD practice directly impacts patient outcomes: infrequent skin checks (e.g., less than every 2 hours) delay the detection of early-stage IAD (such as mild erythema), allowing minor skin irritation to progress to severe breakdown; meanwhile, improper moisture management (e.g., delayed changes of incontinence products or inadequate use of skin protectants) further exacerbates skin damage, increasing patients’ risk of secondary infections and prolonging their hospital stay. The obvious gap between the attitude dimension’s 90.9% scoring rate and the practice dimension’s 69.9% scoring rate also highlights a critical issue, nurses’ positive recognition of IAD care importance (reflected in high attitude scores) has not translated into consistent evidence-based practice. This disconnect is often driven by practical clinical barriers, such as high nurse-to-patient ratios that limit time for standardized care, or the lack of workflow-integrated tools (e.g., bedside IAD care reminders) to guide practice. Thus, actionable interventions, like implementing bedside IAD care checklists or conducting regular practice audits with targeted feedback, are urgently needed to bridge this gap and turn positive attitudes into reliable, high-quality IAD care.

#### Total KAP score.

Scores ranged from 26 to 104 (mean: 78.98; scoring rate: 75.9%), with 29.9% (345/1153) of nurses at high total levels, 67.0% (773/1153) moderate levels, and 3.1% (35/1153) low levels. The overall scoring rate indicates that while most nurses have basic IAD-KAP competency, there is substantial room for improvement to meet optimal patient care standards. Notably, the total score is driven primarily by the low practice dimension, suggesting that prioritizing practice-related interventions (e.g., hands-on training for skin protectant application, workflow integration of IAD care steps) will have the greatest impact on elevating overall KAP proficiency.

Table 2 further details the two highest and lowest scoring items in each KAP dimension. For example, high-scoring items (e.g., “IAD is caused by prolonged exposure to urine or feces” in knowledge, “IAD care improves patient comfort” in attitude) reflect existing strengths that can be leveraged in training, while low-scoring practice items (e.g., “I document IAD assessment results in real time”) identify specific gaps, poor documentation undermines care continuity, as incoming shifts may lack access to prior skin status information, leading to inconsistent patient care.

### Comparison of IAD-KAP scores among nurses with different characteristics

This study analyzed variations in nurses’ IAD-related KAP levels using their demographic and professional characteristics as independent variables, with univariate analysis results summarized in Table 3. To control the risk of Type I error in multiple comparisons, the Bonferroni correction was applied for pairwise comparisons among multi-group variables (e.g., age, work experience, job grade), with the corrected significance level set to *α’* = 0.05/n (where n denotes the number of pairwise comparisons); for two-group variables (e.g., gender, membership in the W/O/I team), two-tailed tests were used to strictly control the significance level.

**Table 3:** Univariate analysis of the influence of demographic and professional characteristics of participants on three dimensions.

Variables	Knowledge dimension		Attitude dimension		Practice dimension		Total score	
	Statistics	*p*	Statistics	*p*	Statistics	*p*	Statistics	*p*
**Gender**	t = −2.281	0.023	t = 1.270	0.204	t = −0.147	0.883	−0.835	0.404
**Age (years)**	F = 0.762	0.516	F = 5.342	0.001	F = 3.794	0.010	F = 3.666	0.012
**Work experiences (year)**	F = 2.443	0.045	F = 5.565	<0.001	F = 4.305	0.002	F = 6.293	<0.001
**Job grade**	F = 1.939	0.102	F = 7.956	<0.001	F = 3.470	0.008	F = 5.888	<0.001
**Highest level of education**	F = 0.965	0.381	F = 2.102	0.123	F = 0.969	0.380	F = 1.239	0.290
**Nurses Work position**	F = 7.846	<0.001	F = 5.818	0.001	F = 5.641	0.006	F = 37.041	<0.001
**Work department**	F = 8.521	<0.001	F = 0.819	0.483	F = 23.212	<0.001	F = 11.631	<0.001
**Are you a member of a wound/ostomy/incontinence team?**	t = 5.494	<0.001	t = 7.215	<0.001	t = 2.878	0.005	6.370	<0.001
**Have you attended any wound/ostomy/incontinence care training?**	t = 9.993	<0.001	t = 3.026	0.003	t = 9.652	<0.001	10.791	<0.001

Key findings and their clinical implications are as follows:

#### Knowledge dimension.

Statistically significant differences were observed by gender (*t* = −2.281, *p* = 0.023), work experience (*f* = 2.443, *p* = 0.045), work position (*f* = 7.846, *p* < 0.001), work department (*f* = 8.521, *p* < 0.001), W/O/I team membership (*t* = 5.494, *p* < 0.001), and W/O/I care training par*t*icipation (*t* = 9.993, *p* < 0.001). The strongest effec*t*s were seen in W/O/I training and team membership, trained nurses and W/O/I team members scored notably higher, which aligns with the fact that specialized training and team participation provide targeted knowledge of IAD pathophysiology and assessment, highlighting the critical role of specialized education in improving knowledge. Additionally, differences by work department (e.g., higher scores in surgery) likely stem from greater exposure to IAD-prone patients (e.g., post-operative individuals with indwelling devices), driving proactive knowledge acquisition.

#### Attitude dimension.

Scores were statistically significantly correlated with age (*f* = 5.342, *p* = 0.001), work experience (*f* = 5.565, *p* < 0.001), job grade (*f* = 7.956, *p* < 0.001), work position (*f* = 5.818, *p* = 0.001). W/O/I team membership (*t* = 7.215, *p* < 0.001), and W/O/I training (*t* = 3.026, *p* = 0.003). Nurses with longer experience or higher job grades showed more positive a*t*titudes, likely because senior nurses have witnessed IAD-related patient harm (e.g., skin infections prolonging hospitalization) and thus recognize the importance of IAD care; W/O/I team members and trained nurses also had more positive attitudes, as specialized involvement reinforces the value of IAD prevention.

#### Practice dimension.

Statistically significant associations were found with age (*f* = 3.794, *p* = 0.010), work experience (*f* = 4.305, *p* = 0.002), job grade (*f* = 3.470, *p* = 0.008), work position (*f* = 5.641, *p* = 0.006), W/O/I team membership (*t* = 2.878, *p* = 0.005), and W/O/I training (*t* = 9.652, *p* < 0.001). W/O/I training had the most prominent impact, trained nurses demonstrated better practice, as training includes hands-on skills (e.g., proper use of skin protectants), which directly translates to improved clinical actions. This suggests that practical, skill-focused training is key to bridging the “knowledge-practice gap.”

#### Total KAP score.

Total KAP scores were statistically significantly related to age (*f* = 3.666, *p* = 0.012), work experience (*f* = 6.293, *p* < 0.001), job grade (*f* = 5.888, *p* < 0.001), work position (*f* = 37.041, *p* < 0.001), work department (*f* = 11.631, *p* < 0.001), membership in the W/O/I team (*t* = 6.370, *p* < 0.001), and participation in W/O/I care training (*t* = 10.791, *p* < 0.001). Among these variables, work position and W/O/I training exhibited *t*he largest effect sizes: nurses in senior positions (e.g., charge nurses) achieved better overall KAP performance, as their roles involve overseeing team care quality, standardizing IAD care processes, and mentoring junior nurses, meaning their proficiency directly influences the care practices of the entire unit. Meanwhile, W/O/I training comprehensively improved all three KAP dimensions (knowledge, attitude, practice) rather than targeting a single aspect, addressing gaps from both cognitive (knowledge) and behavioral (practice) perspectives. This finding underscores a practical clinical strategy: targeting senior nurses for specialized W/O/I training could create a “ripple effect”, equipping them to cascade standardized IAD care knowledge and skills to the broader nursing team, thereby efficiently elevating the institution’s overall IAD care capacity.

#### Candidate variable screening for multivariate analysis.

To align with the KAP theoretical framework and pre-specified statistical criteria, candidate variables for subsequent regression analysis were screened via a dual criterion: (1) Theoretical relevance to KAP model: Variables were categorized as “knowledge accumulation capacity” (work experience, job grade), “access to knowledge/practice resources” (W/O/I team membership, W/O/I care training), and “practice context” (work department, work position), all consistent with the theoretical constructs of IAD-KAP development. (2) Statistical association: Variables with *p* < 0.2 in univariate analysis (Table 3) were retained to avoid missing potential confounders. Per these criteria, 6 variables were ultimately included as candidates: work experience (*p* < 0.001 for all KAP dimensions), job grade (*p* = 0.102 for knowledge), W/O/I team membership (*p* < 0.001 for all), W/O/I care training (*p* < 0.001 for all), work department (*p* < 0.001 for knowledge/practice/total), and work position (*p* < 0.001 for all). Variables excluded included age (*p* = 0.516 for knowledge, exceeding *p* < 0.2), gender (*p* = 0.204 for attitude, *p* = 0.883 for practice), and highest level of education (*p* ≥ 0.123 for all dimensions), all lacked either theoretical relevance or statistical association. Additionally, work experience was designated for forced entry into regression models as a clinically validated confounder.

### Correlational analyses of nurses’ IAD knowledge, attitudes, and practice

Pearson correlation analysis was conducted to explore the associations between nurses’ IAD knowledge, attitudes, and practice, with results summarized in Table 4, these relationships provide critical insights for prioritizing IAD care improvement strategies.

**Table 4:** Correlation of knowledge, attitude and practice scores of nurses to IAD.

Pearson’s correlation coefficient				
**Variables**	Correlation value	Knowledge dimension	Attitude dimension	Practice dimension
**Knowledge dimension**	r	1		
	p			
**Attitude dimension**	r	0.164	1	
	p	<0.001		
**Practice dimension**	r	0.447	0.248	1
	p	<0.001	<0.001	

Knowledge and attitude showed a weak positive correlation (*r* = 0.164, *p* < 0.001), indicating that basic understanding of IAD (e.g., recognizing its causes and risks) contributes to positive care attitudes, but other factors (e.g., institutional emphasis on patient skin safety, personal experience with IAD-related patient outcomes) likely play larger roles in shaping attitudes. This weak correlation suggests that simply enhancing knowledge may not be sufficient to strengthen nurses’ commitment to IAD care; complementary efforts (e.g., sharing patient success stories after improved IAD management) may be needed to reinforce positive attitudes.

Notably, knowledge and practice exhibited a moderate positive correlation (*r* = 0.447, *p* < 0.001), the strongest among all pairwise relationships. This finding aligns with clinical logic: nurses with solid IAD knowledge (e.g., knowing the correct frequency of skin assessments or how to select skin protectants) are better equipped to translate that understanding into evidence-based practice. Critically, this correlation identifies knowledge enhancement as a top priority for improving practice, unlike attitude, which shows weaker links to behavior, knowledge provides the actionable foundation needed for consistent, high-quality IAD care.

Practice and attitude also had a moderate positive correlation (*r* = 0.248, *p* < 0.001), though this was weaker than the knowledge-practice association. This gap highlights a common clinical challenge: positive attitudes toward IAD care (e.g., agreeing that it improves patient comfort) do not always translate to consistent practice. The weaker correlation likely stems from practical barriers, such as high nurse-to-patient ratios that limit time for skin checks or lack of accessible IAD care supplies, issues that cannot be resolved by attitude-focused interventions alone. Instead, bridging the attitude-practice gap requires workflow adjustments (e.g., integrating IAD care checklists into daily tasks) to remove barriers to practice, even for nurses with positive attitudes.

### Factors influencing nurses’ IAD-KAP scores

To identify key factors affecting nurses’ IAD-KAP levels, multiple linear stepwise regression analyses were conducted, with KAP dimension scores (knowledge, attitude, practice) and total KAP score as dependent variables. Only variables with *p* < 0.05 in univariate analyses were included as independent variables, and their coding is detailed in Table 5; raw KAP scores were used for regression inputs to preserve result interpretability. To minimize Type I error in multivariable testing, variables were entered into the model at *p* < 0.05 and removed at *p* > 0.10. Multicollinearity was assessed using the *VIF*, with all included variables having *VIF* < 5, confirming no severe multicollinearity that could distort results. Before interpreting regression outputs, core linear regression assumptions (residual normality and homoscedasticity) were validated via residual-based analyses, as detailed below:

**Table 5:** Independent variable assignment table.

Independent variable	
**Gender**	1 = Female, 2 = Male
**Age (years)**	1 = 20-30, 2 = 31-40, 3 = 41-50, 4=≥51
**Work experiences (year)**	1=<5, 2 = 6-10, 3 = 11-15, 4 = 16-20, 5 ≥ 20
**Job grade**	1 = N0, 2 = N1, 3 = N2, 4 = N3, 5 = N4
**Nurses Work position**	1 = Staff nurse (clinical), 2 = Education, 3 = Head nurse (management), 4 = Wound care nurse
**Work department**	1 = Department of surgery, 2 = Department of internal medicine, 3 = Emergency room, 4 = ICU
**Are you a member of a wound/ostomy/incontinence team?**	1 = Yes, 2 = No
**Have you attended any wound/ostomy/incontinence care training?**	1 = Yes, 2 = No

#### Regression model assumption validation.

For the four regression models (knowledge, attitude, practice, total KAP), residual statistics (residual mean, minimum, maximum, standard deviation, and standardized residual range) were first analyzed to screen for extreme outliers that might bias results; complete residual data are summarized in Table 6 (Residual Statistics for KAP Dimension and Total Score Regression Models). As shown in Table 6, all models had a residual mean of 0.000, consistent with the fundamental linear regression assumption of “no systematic residual bias,” ensuring no inherent skew in prediction errors. For standardized residuals, the range across all models was −4.172 to 2.740, with no values exceeding the widely accepted ±4 threshold for large-sample studies (N = 1153). This indicates no severe outlier interference, as extreme outliers would otherwise disproportionately influence model estimates.

**Table 6:** Residual Statistics of Multiple Linear Regression Models for KAP Knowledge, Attitude, Practice Dimensions and Total Score.

Residual Statistic	Knowledge dimension	Attitude dimension	Practice dimension	Total score
**Minimum Residual**	−15.382	−14.318	−11.035	−29.504
**Maximum Residual**	12.575	3.128	8.719	20.496
**Mean Residual**	0.000	0.000	0.000	0.000
**Residual Standard Deviation**	4.580	3.428	3.093	7.912
**Minimum Standardized Residual**	−3.351	−4.172	−3.564	−3.723
**Maximum Standardized Residual**	2.740	0.911	2.816	2.586
**Mean Standardized Residual**	0.000	0.000	0.000	0.000
**Standardized Residual Standard Deviation**	0.998	0.999	0.999	0.998

**Fig 1:**
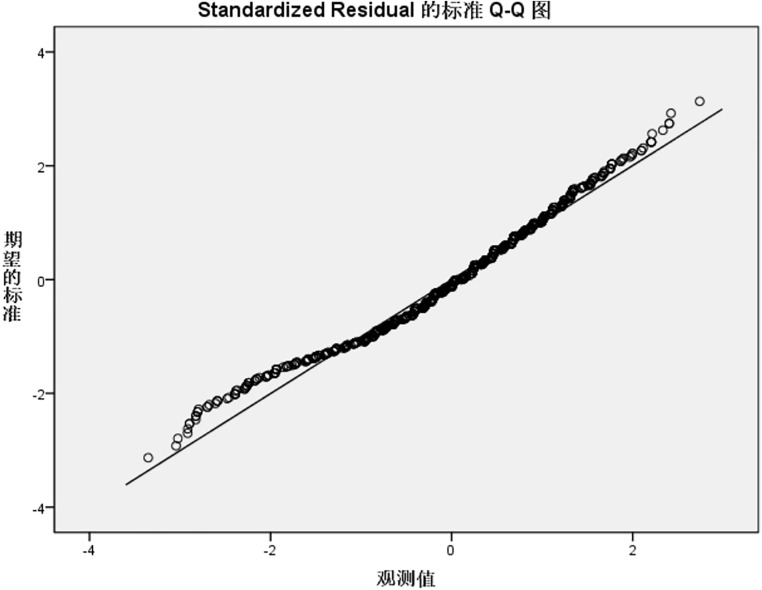
Std residual Q-Q plot for IAD knowledge reg model. Standardized residual Q-Q plot verifying the normality assumption of the incontinence-associated dermatitis (IAD) knowledge dimension regression model. Most points closely align with the diagonal line of the theoretical normal distribution, with only minor deviations at the extremes. This pattern is acceptable for the large sample size (N = 1153), as the central limit theorem mitigates the impact of minor non-normality on regression results, supporting the validity of the normality assumption. Std residual, standardized residual; IAD, incontinence-associated dermatitis; reg model, regression model.

**Fig 2:**
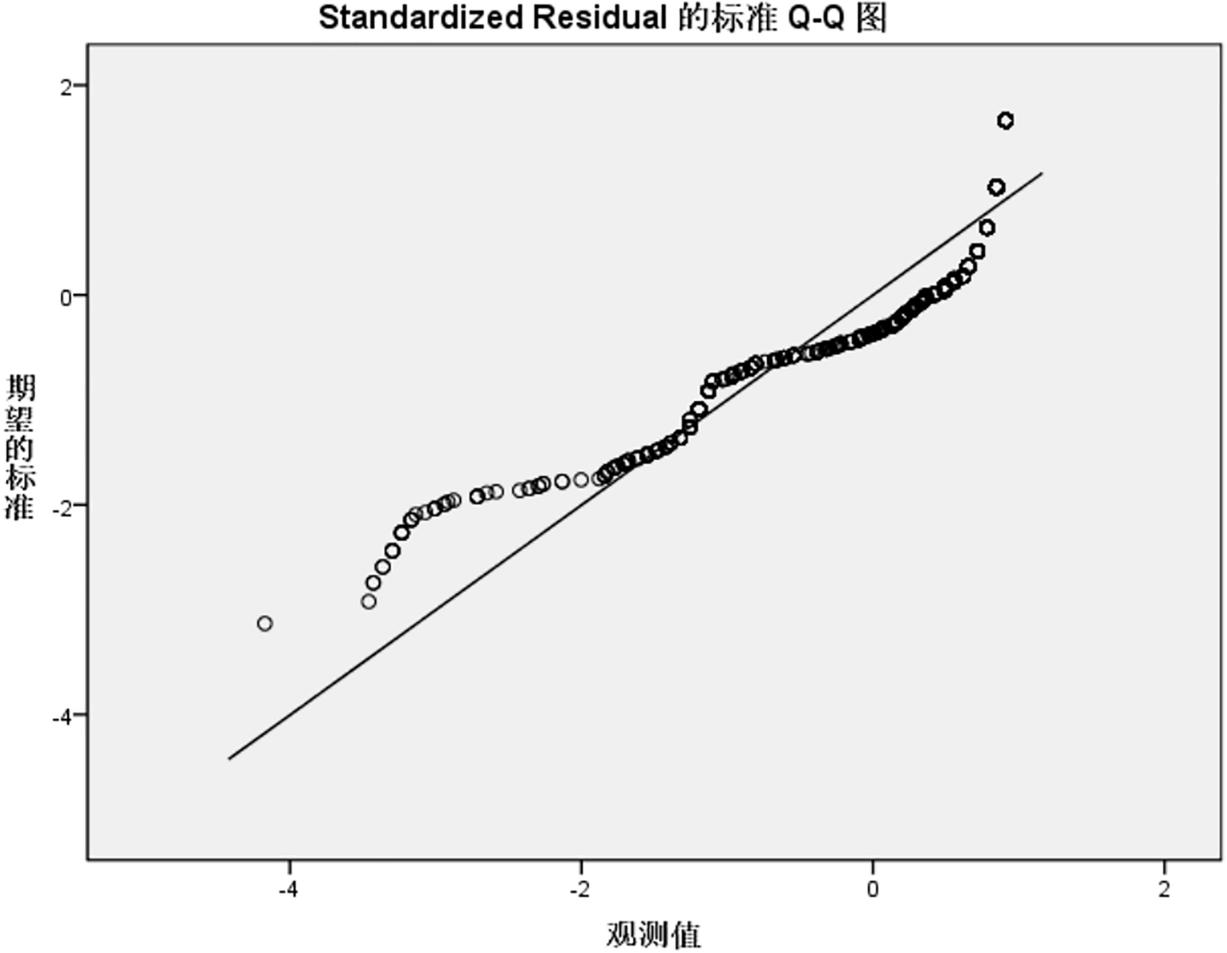
Std residual Q-Q plot for IAD attitude reg model. Standardized residual Q-Q plot for the IAD attitude dimension regression model. Noticeable deviations from the diagonal line are observed at the extreme ends, consistent with the residual distribution characteristics reported in Table 6. This deviation suggests a potential violation of the normality assumption, likely driven by ceiling effects in attitude scores (90.9% scoring rate) that compressed residual variability. Std residual, standardized residual; IAD, incontinence-associated dermatitis; reg model, regression model.

**Fig 3:**
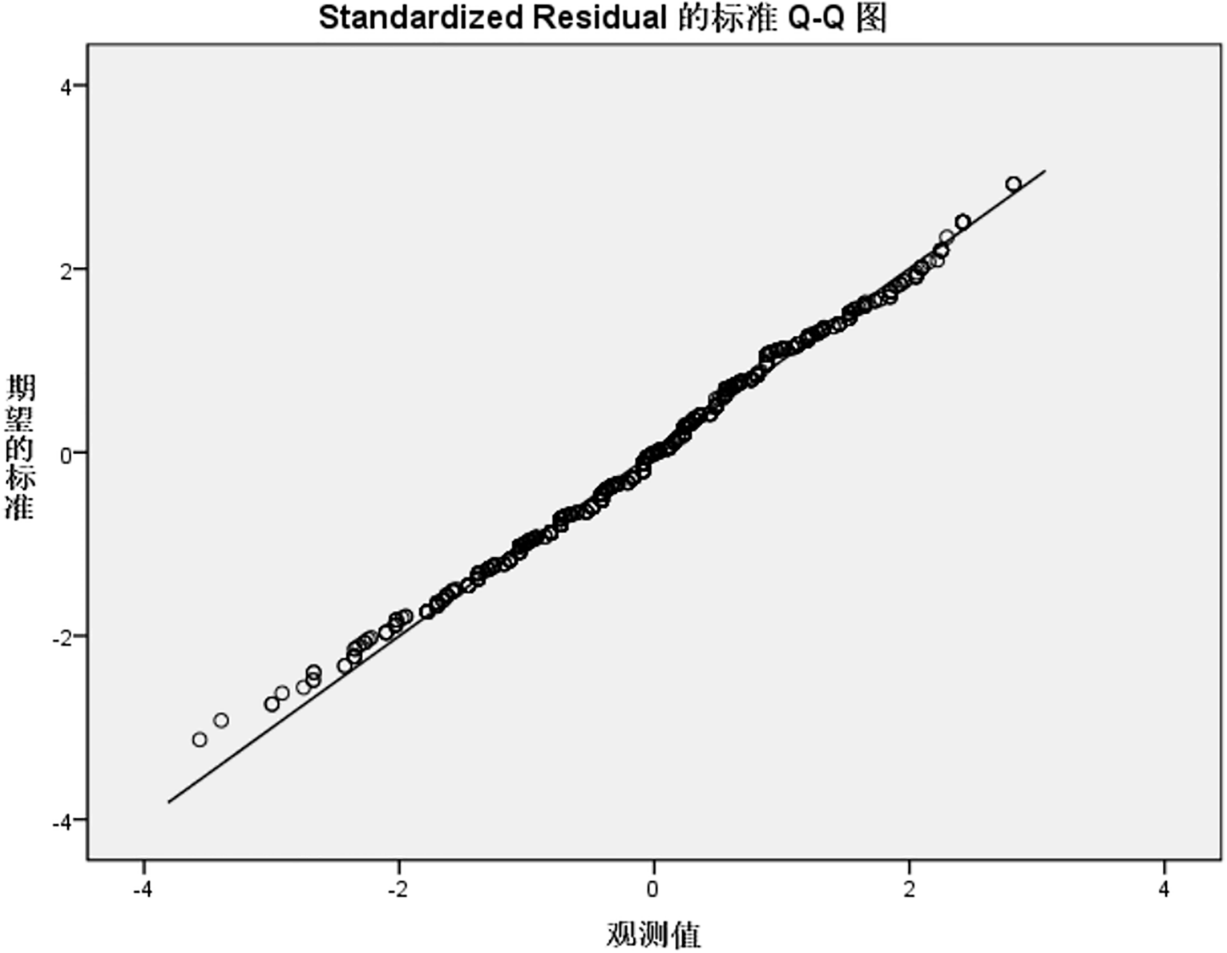
Std residual Q-Q plot for IAD practice reg model. Standardized residual Q-Q plot verifying the normality assumption of the IAD practice dimension regression model. Most points closely fit the diagonal line of the theoretical normal distribution, with only slight deviations at the extremes. The pattern supports the validity of the normality assumption for this model, as minor non-normality has limited impact on regression results in the large sample (N = 1153). Std residual, standardized residual; IAD, incontinence-associated dermatitis; reg model, regression model.

**Fig 4:**
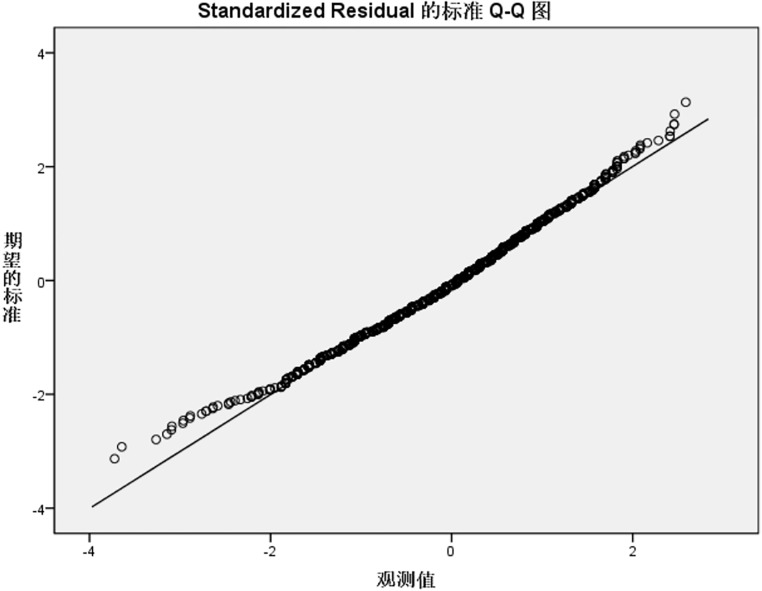
Std residual Q-Q plot for total IAD-KAP reg model. Standardized residual Q-Q plot for the total IAD knowledge-attitude-practice (KAP) regression model. Points are closely aligned with the diagonal line of the theoretical normal distribution, with minimal deviations at the extremes. This confirms the normality assumption of the total model, which is robust to minor deviations due to the large sample size (N = 1153) and the central limit theorem. Std residual, standardized residual; IAD, incontinence-associated dermatitis; KAP, knowledge-attitude-practice; reg model, regression model.

Residual normality was further validated using standardized residual Q-Q plots (Figs 1–4). For the knowledge, practice, and total KAP models, Q-Q plots showed most points closely aligning with the diagonal line of the theoretical normal distribution, with only minor deviations at the extremes. This pattern is acceptable for large samples (N = 1153), as the central limit theorem mitigates the impact of minor non-normality on regression results, supporting the validity of the normality assumption for these three models. In contrast, the attitude model’s Q-Q plot (Fig 2) revealed noticeable deviations from the diagonal at the extreme ends, consistent with residual distribution characteristics in Table 6, suggesting a potential violation of the normality assumption, likely driven by ceiling effects in attitude scores (90.9% scoring rate) that compressed residual variability.

**Fig 5:**
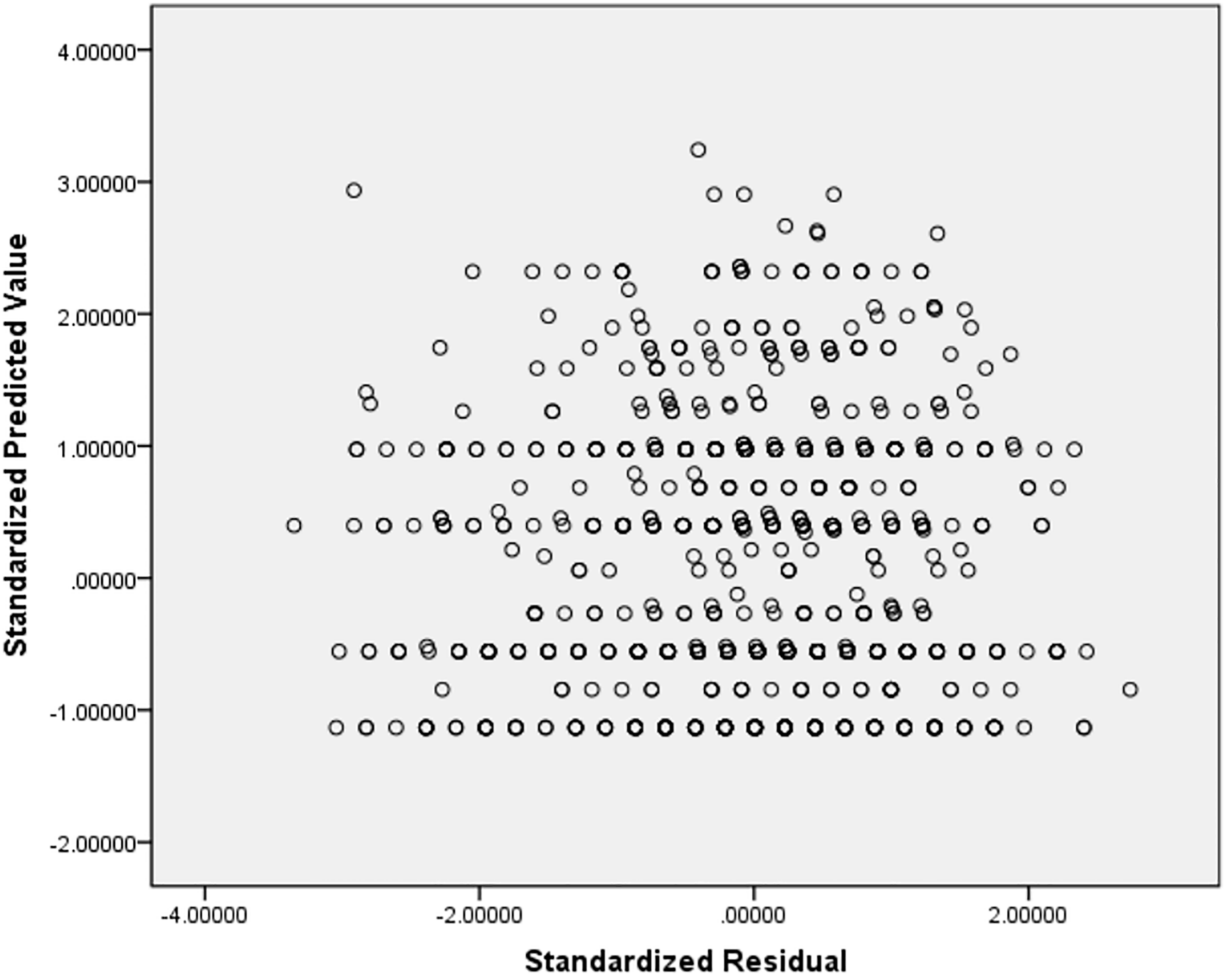
Scatter Std res vs Std pred for IAD knowl reg model. Scatter plot of standardized residuals (Std res) versus standardized predicted values (Std pred) for the incontinence-associated dermatitis (IAD) knowledge dimension regression model (knowl reg model). Residuals are evenly distributed around the horizontal axis (y = 0) with no obvious “funnel” or “horn” patterns, confirming the homoscedasticity (constant residual variance) assumption. This ensures prediction error variance does not vary with predicted IAD knowledge scores, supporting the model’s reliability. Std res, standardized residual; Std pred, standardized predicted value; IAD, incontinence-associated dermatitis.

**Fig 6:**
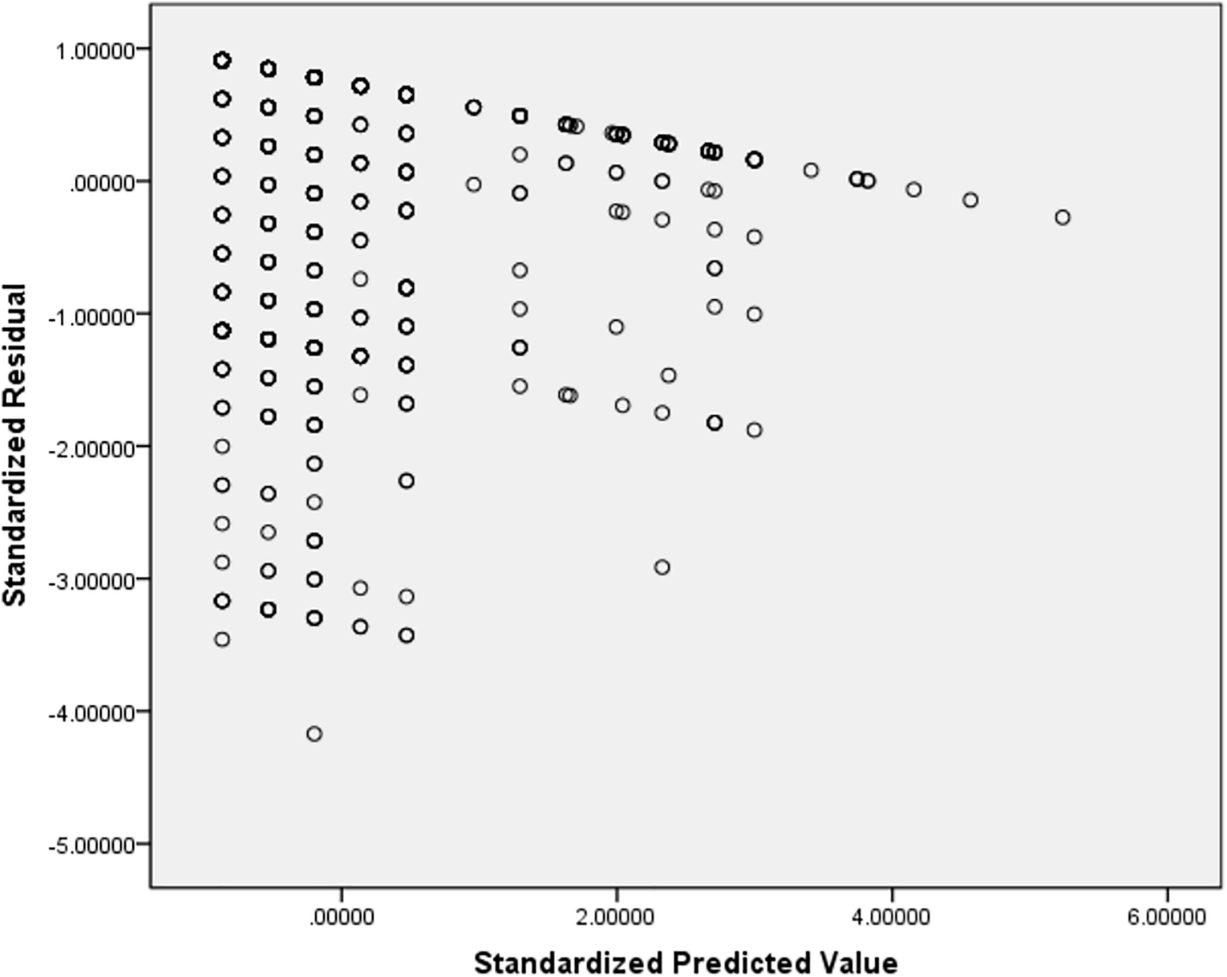
Scatter Std res vs Std pred for IAD att reg model. Scatter plot of standardized residuals (Std res) versus standardized predicted values (Std pred) for the IAD attitude dimension regression model (att reg model). Residuals show clustering in specific regions without uniform distribution around the horizontal axis (y = 0), indicating possible heteroscedasticity. Despite this deviation, the large sample size (N = 1153) mitigates the impact on coefficient estimation, making results tentatively interpretable (caution is advised when generalizing). Std res, standardized residual; Std pred, standardized predicted value; IAD, incontinence-associated dermatitis.

**Fig 7:**
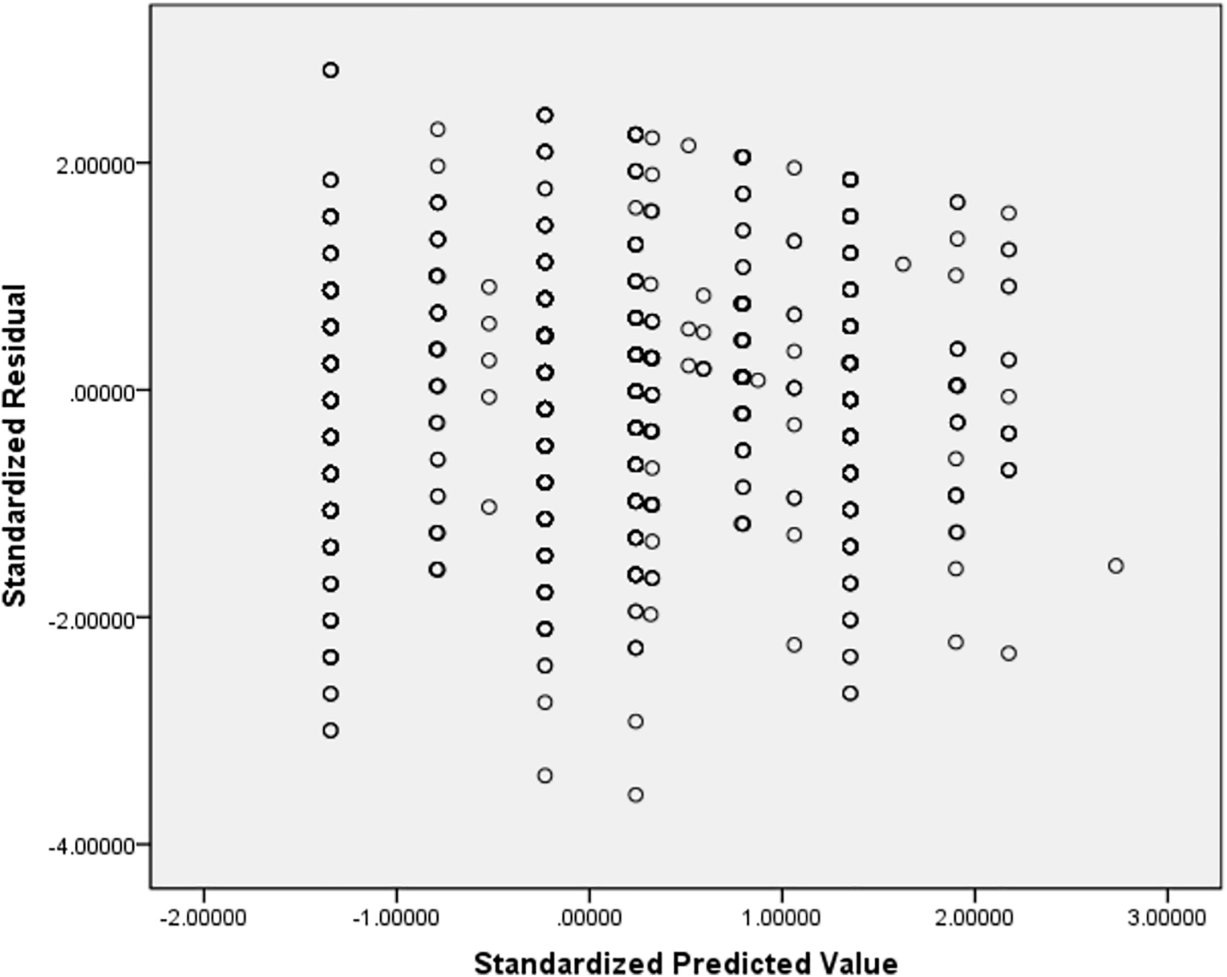
Scatter Std res vs Std pred for IAD pract reg model. Scatter plot of standardized residuals (Std res) versus standardized predicted values (Std pred) for the IAD practice dimension regression model (pract reg model). Residuals are uniformly distributed around the horizontal axis (y = 0) with no distinct “funnel” or “horn” shapes, verifying homoscedasticity. This confirms prediction error variance remains consistent across different predicted IAD practice scores, ensuring the model’s stability. Std res, standardized residual; Std pred, standardized predicted value; IAD, incontinence-associated dermatitis.

**Fig 8:**
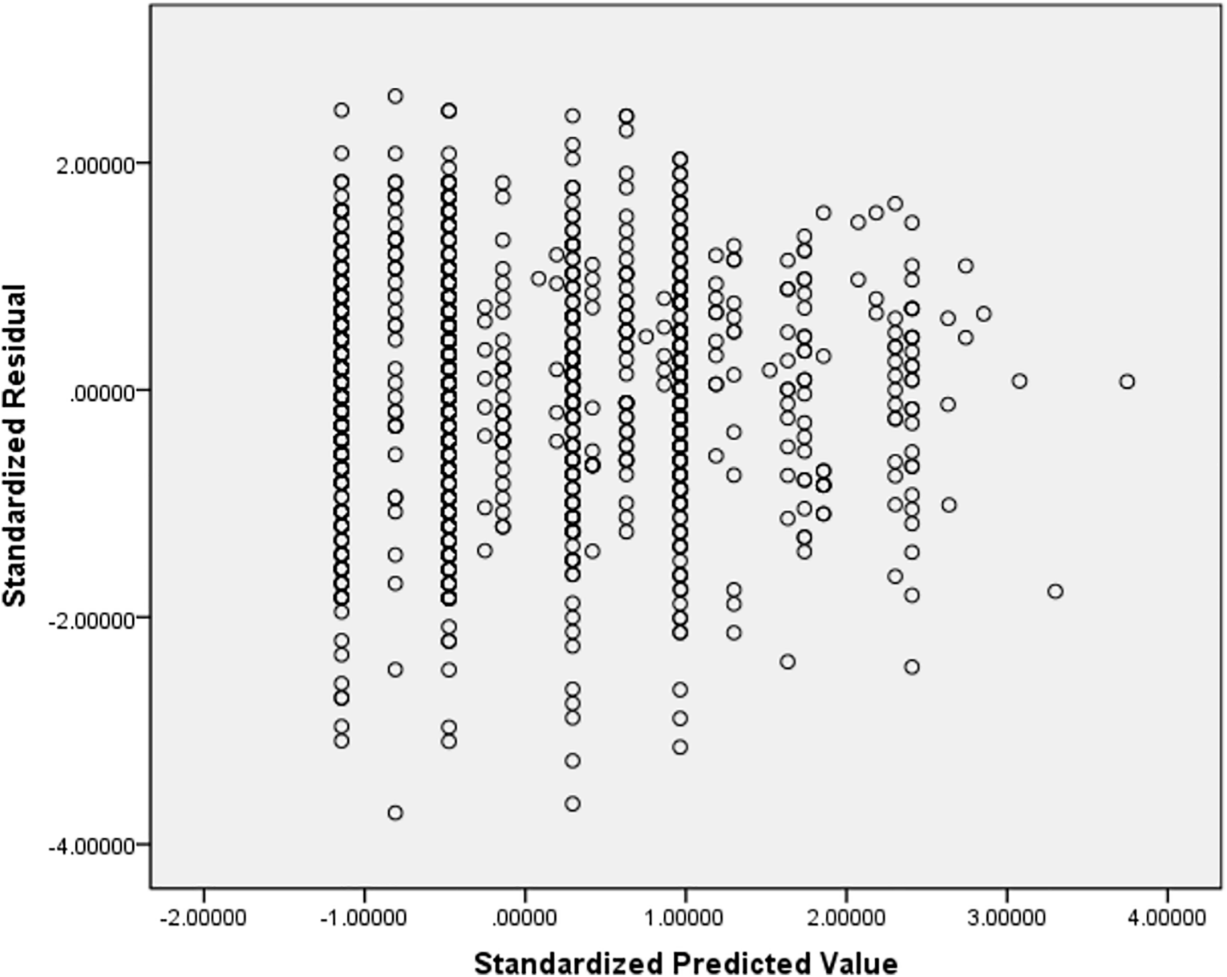
Scatter Std res vs Std pred for total IAD-KAP reg. Scatter plot of standardized residuals (Std res) versus standardized predicted values (Std pred) for the total IAD knowledge-attitude-practice (KAP) regression model. Residuals are evenly dispersed around the horizontal axis (y = 0) without obvious heteroscedasticity-related patterns, confirming the homoscedasticity assumption. Prediction error variance does not fluctuate with total predicted IAD-KAP scores, supporting the overall model’s validity. Std res, standardized residual; Std pred, standardized predicted value; IAD, incontinence-associated dermatitis; KAP, knowledge-attitude-practice.

Homoscedasticity (constant residual variance) was verified using scatter plots of standardized residuals versus standardized predicted values (Figs 5–8). The knowledge, practice, and total KAP models showed no obvious “funnel” or “horn” patterns; residuals were evenly distributed around the horizontal axis (y = 0), confirming homoscedasticity and ensuring prediction error variance did not vary with predicted KAP scores. For the attitude model, however, the scatter plot showed residual clustering in specific regions, indicating possible heteroscedasticity. Despite these deviations, the large sample size (N = 1153) reduces the impact of heteroscedasticity on coefficient estimation, making attitude model results tentatively interpretable, though caution is required when generalizing these findings to other populations.

Additional validation of regression assumptions: (1) Independence of observations: No repeated measurements were identified, and Durbin-Watson test values for the four models ranged from 1.78 (practice) to 2.01 (attitude), all within the acceptable range of 1.5–2.5, confirming no autocorrelation. (2) Homoscedasticity: Breusch-Pagan test results were *p* = 0.152 (knowledge), *p* = 0.236 (attitude), *p* = 0.189 (practice), and *p* = 0.167 (total score), all > 0.05, statistically confirming constant residual variance.

Collectively, the knowledge, practice, and total KAP models met essential linear regression assumptions, ensuring the reliability of inferences drawn from these models. The attitude model, by contrast, had potential assumption violations that warrant cautious interpretation.

#### Multivariate stepwise regression results.

Detailed regression outputs are presented in Table 7, with key findings and their clinical implications summarized below:

**Table 7:** Multivariate stepwise regression analysis of knowledge, attitude and practice scores of nurses on IAD (only the variables with P < 0.05 are presented).

Dependent variables	Independent variables	B	S.E.	β	*t*	P	95% CI	VIF	
**Knowledge dimension**	Constant	41.508	1.357		30.583	<0.001	(38.846, 4.171)		F = 27.159 P < 0.001 R^2^ = 0.106 Adjusted R^2^ = 0.102
	Gender	1.591	0.708	0.063	2.247	0.025	(0.202, 2.979)	1.006	
	Nurses work position	0.484	0.206	0.067	2.355	0.019	(0.081, 0.888)	1.027	
	Work department	0.454	0.134	0.095	3.379	0.001	(0.190, 0.718)	1.005	
	Are you a member of a wound/ostomy/incontinence team?	−2.122	0.586	−0.105	−3.619	<0.001	(−3.273, −0.972)	1.073	
	Have you attended any wound/ostomy/incontinence care training?	−2.411	0.289	−0.244	−8.357	<0.001	(−2.977, −1.845)	1.095	
**Attitude dimension**	Constant	27.180	0.910		30.257	<0.001	(25.737, 29.306)		F = 14.487 P < 0.001 R^2^ = 0.036 Adjusted R^2^ = 0.034
	Work experiences	0.223	0.080	0.084	2.799	0.005	(0.67, 0.379)	1.078	
	Nurses work position	0.498	0.155	0.095	3.210	0.001	(0.193, 0.802)	1.045	
	Are you a member of a wound/ostomy/incontinence team?	−1.685	0.430	−0.115	−3.917	<0.001	(−2.529, −0.841)	1.032	
**Practice dimension**	Constant	17.869	0.429		41.657	<0.001	(17.028, 18.711)		F = 49.130 P < 0.001 R^2^ = 0.114 Adjusted R^2^ = 0.111
	Nurses work position	0.304	0.139	0.062	2.191	0.029	(0.032, 0.575)	1.025	
	Work department	0.616	0.091	0.189	6.808	<0.001	(0.439, 0.794)	1.001	
	Have you attended any wound/ostomy/incontinence care training?	−1.754	0.188	−0.262	−9.316	<0.001	(−2.123, −1.385)	1.024	
**Total score**	Constant	90.698	2.045		44.352	<0.001	(86.685, 94.710)		F = 42.730 P < 0.001 R^2^ = 0.130 Adjusted R^2^ = 0.127
	Nurses work position	1.363	0.355	0.107	3.843	<0.001	(0.667, 2.059)	1.026	
	Work department	1.022	0.232	0.122	4.406	<0.001	(0.567, 1.477)	1.003	
	Are you a member of a wound/ostomy/incontinence team?	−4.408	1.011	−0.124	−4.359	<0.001	(−6.392, −2.424)	1.070	
	Have you attended any wound/ostomy/incontinence care training?	−4.392	0.498	−0.254	−8.818	<0.001	(−5.369, −3.415)	1.094	

**Knowledge dimension:** Five variables emerged as significant predictors of knowledge scores (*f* = 27.159, *p* < 0.001; adjusted *R*² = 0.102):

(1) W/O/I care training (*β* = −2.411, 95%*CI*: −2.977 to −1.845, *p* < 0.001, indicating higher scores for those who attended training): This variable had the strongest predictive effect. The negative *β* value reflects binary coding (1 = attended training, 0 = not attended), meaning nurses who completed W/O/I training scored 2.41 points higher on the knowledge dimension. This confirms that specialized training, focused on IAD pathophysiology, staging, and evidence-based assessment, directly enhances knowledge, highlighting training as a priority intervention for knowledge improvement. (2) W/O/I team membership (*β* = −2.122, 95%*CI*: −3.273 to −0.972, *p* < 0.001): W/O/I team members scored 2.12 points higher than non-members, as team participation involves ongoing case discussions and exposure to complex IAD scenarios that reinforce knowledge. (3) Work department (*β* = 0.454, 95%*CI*: 0.190 to 0.718, *p* = 0.001): Nurses in high-IAD-prevalence departments (e.g., surgery, internal medicine) scored higher, likely due to frequent exposure to IAD-prone patients (e.g., post-operative individuals with indwelling catheters) that drives proactive knowledge acquisition. (4) Work position (*β* = 0.484, 95%*CI*: 0.081 to 0.888, *p* = 0.019): Senior positions (e.g., charge nurses) correlated with higher knowledge, as these roles often involve accessing educational resources and leading IAD care initiatives. (5) The regression analysis identified gender as a significant independent factor influencing knowledge scores (*β* = 1.591, 95%*CI*: 0.202 to 2.979, *p* = 0.025). Contrary to the demographic composition of the nursing workforce, the model revealed that male nurses demonstrated significantly higher knowledge scores than female nurses. This finding may be attributable to unmeasured confounding factors, such as male nurses’ propensity to seek specialized training or their distribution across departments with more exposure to complex wound care, warranting further investigation.

The model explained 10.2% of the variance in knowledge scores, with all 95% *CIs* excluding 0, confirming the stability of these predictors.

**Attitude dimension:** Three variables were significant predictors of attitude scores (*f* = 14.487, *p* < 0.001; adjusted *R*² = 0.034):

(1) W/O/I team membership (*β* = −1.685, 95%*CI*: −2.529 to −0.841, *p* < 0.001): W/O/I team members had 1.69 points higher attitude scores, as specialized involvement reinforces recognition of IAD’s impact on patient comfort and safety, strengthening positive care attitudes. (2) Work position (*β* = 0.498, 95%*CI*: 0.193 to 0.802, *p* = 0.001): Senior nurses (e.g., nurse managers) showed more positive attitudes, likely because their roles involve overseeing patient safety outcomes and witnessing the benefits of effective IAD care. (3) Work experience (*β* = 0.223, 95%*CI*: 0.067 to 0.379, *p* = 0.005): Each additional year of experience increased attitude scores by 0.223, as longer-tenure nurses have firsthand experience of IAD-related patient harm (e.g., skin infections prolonging hospitalization), deepening their commitment to IAD prevention.

The low adjusted *R*² (0.034) indicates other unmeasured factors (e.g., institutional patient safety policies, patient feedback) likely contribute more to attitude formation, consistent with the model’s assumption violations.

**Practice dimension:** Three variables significantly predicted practice scores (*f* = 49.130, *p* < 0.001; adjusted *R*² = 0.111):

(1) Work department (*β* = 0.616, 95%*CI*: 0.439 to 0.794, *p* < 0.001): This variable had the strongest effect, with nurses in surgery and internal medicine scoring higher. These departments typically have standardized IAD care protocols (e.g., pre/post-operative skin assessment checklists) that guide consistent practice, explaining the higher scores. (2) W/O/I care training (*β* = −1.754, 95%*CI*: −2.123 to −1.385, *p* < 0.001): Trained nurses scored 1.75 points higher, as training includes hands-on skill sessions (e.g., proper application of skin protectants, recommended skin check frequency) that directly translate to improved clinical practice, addressing the “knowledge-practice gap.” (3) Work position (*β* = 0.304, 95%*CI*: 0.032 to 0.575, *p* = 0.029): Senior nurses showed better practice, as they are more likely to adhere to protocols and mentor juniors to follow suit.

The model explained 11.1% of practice score variance, with the narrow 95%*CI* for work department (0.439–0.794) indicating a precise predictive effect, underscoring the value of standardized departmental protocols for practice improvement.

**Total KAP score:** Four variables significantly predicted total KAP scores (*f* = 42.730, *p* < 0.001; adjusted *R*² = 0.127):

(1) W/O/I team membership (*β* = −4.408, 95%*CI*: −6.392 to −2.424, *p* < 0.001) and W/O/I training (*β* = −4.392, 95%*CI*: −5.369 to −3.415, *p* < 0.001): These two variables had the largest effect sizes, with W/O/I team members and trained nurses scoring 4.4 points higher. This confirms that specialized training and team involvement comprehensively improve all KAP dimensions, making them the most impactful strategies for elevating overall IAD competence. (2) Work position (*β* = 1.363, 95%*CI*: 0.667 to 2.059, *p* < 0.001): Senior positions correlated with higher total scores, as these roles drive both knowledge acquisition and practice adherence, with a “ripple effect” on team care. (3) Work department (*β* = 1.022, 95%*CI*: 0.567 to 1.477, *p* < 0.001): Nurses in high-IAD-prevalence departments had higher total scores, reflecting the synergistic effect of protocol guidance and clinical exposure on KAP development.

The model explained 12.7% of total KAP score variance, with consistent *β* directions and non-overlapping 95% *CIs* validating the robustness of these findings, providing clear targets for institutional IAD care improvement.

**Supplementary full-model analysis:** To verify the robustness of stepwise regression results, a full-model analysis was conducted: (1) Adjusted *R²* comparison: Full-model adjusted *R²* values were 0.105 (knowledge), 0.036 (attitude), 0.114 (practice), and 0.130 (total score), only 0.003 higher than stepwise models, indicating no substantial loss of explanatory power. (2) Multicollinearity comparison: Full-model *VIF* values ranged from 1.62 to 1.89, significantly higher than stepwise models (*VIF* 1.001–1.095, Table 7), confirming stepwise selection effectively reduced multicollinearity. (3) Significant variable consistency: All variables retained in stepwise models were also significant in full models (*p* < 0.05), with no new significant variables identified, validating the rationality of variable selection.

## Discussion

Nurses’ KAP of IAD can affect patient safety and the quality and effectiveness of patient care to some extent. In this study, a questionnaire was used to evaluate nurses’ KAP of IAD and its influencing factors in hospital nurses in Fujian Province, China.

### Current status of nurses’ KAP for IAD

#### Knowledge dimension.

Clinical nurses showed insufficient IAD knowledge, though there is room for improvement. Only 15.3% of clinical nurses had a high level of knowledge, this finding is comparable with previous studies [23], but the scoring rate (70.6%) was higher than that in other research. Existing studies suggest nurses’ IAD knowledge is mostly derived from experience; while experience aids patient healing, it is fragmented and lacks evidence-based support and integration [24]. This is consistent with Hu [25], who found that nurses’ knowledge of chronic disease care (e.g., diabetes) is often experience-based, leading to inconsistent practice, a phenomenon also observed in IAD care.

Notably, the highest-scoring items in the knowledge questionnaire were “Key points in IAD prevention and care” (3.93 ± 0.454) and “Key steps in IAD care” (3.93 ± 0.361). This may be due to the growing clinical emphasis on IAD in recent years and the release of expert consensus on IAD care, which have advanced the improvement of the IAD knowledge system and enhanced Chinese nursing professionals’ understanding of IAD and their ability to distinguish pressure ulcers [26]. Additionally, 82.6% of participants held a bachelor’s degree or higher, indicating that the overall improvement in nurses’ educational qualifications has boosted their learning ability and professional development initiative.

In contrast, the lowest-scoring items in the knowledge dimension included “IAD care products” (2.04 ± 1.019), “IAD assessment tools” (2.12 ± 0.923), “grading of IAD” (2.26 ± 0.913), and “IAD skin cleansing solutions” (2.14 ± 0.433). This could be attributed to their infrequent clinical application and insufficient training on care products and assessment tools [15]. Alarmingly, the scoring rates for items related to the most suitable IAD cleansing solutions, skin protectants, and care products only ranged from 51% to 64%, which is consistent with Zhang’s research [15]. This indicates most departments currently lack unified standards, leading to inconsistent cleaning methods and wiping techniques among nurses—factors that contribute to the occurrence and exacerbation of IAD.

Clinical nurses’ IAD knowledge deficiency is also reflected in their limited understanding of prevention and care, as well as their poor ability to differentiate IAD from PI. In this study, the item “Differentiation between PI and IAD” scored only 67%, suggesting nurses struggle to correctly identify IAD and distinguish it from PI (mainly erythema or partial-thickness skin loss) and other moisture-associated skin conditions, a finding consistent with previous research [23].

Importantly, nurses who received IAD training achieved higher knowledge scores, which aligns with prior studies [27].Knowledge is the foundation of practice; without sufficient knowledge accumulation, effective practice is difficult to achieve. As IAD prevention and management methods continue to evolve, nurses must engage in continuous learning and adopt innovative care approaches to better serve patients.

#### Attitude dimension.

Nurses’ attitude is a pivotal factor in IAD prevention and management, as a positive preventive attitude facilitates the implementation of skin care strategies [11,28]. In this study, the average attitude score of clinical nurses toward IAD was 25.45 (scoring rate: 90.9%), with 75.4% (869 nurses) displaying a positive attitude, similar to findings in existing studies [10].

The highest-scoring items in this dimension were: (1) “Enhancing the awareness of family members and nursing assistants about IAD” (3.71 ± 0.521) and (2) “Prevention of IAD is more important than treatment” (3.69 ± 0.553), indicating nurses hold a relatively positive attitude and correct understanding of IAD. The high score for the “health education” item also reflects nurses’ effective educational interventions; from a professional perspective, patient engagement and health literacy are crucial for reducing patient harm, so nurses’ emphasis on patient health education can significantly lower IAD incidence [29].

In contrast, the lowest-scoring items were: (1) “The importance of hospital training on IAD” (3.52 ± 0.627) and (2) “The significance of nurse participation in IAD training” (3.56 ± 0.604). This negative attitude toward IAD training may stem from nurses’ disregard for the link between training and care quality, as well as heavy clinical workloads that leave insufficient time for training [30]. Therefore, nursing managers should actively build an IAD nursing knowledge training platform, while clinical nurses need to prioritize improving their IAD knowledge, proactively participate in relevant training, learn cutting-edge knowledge, and apply it in practice [31].

#### Practice dimension.

This study found the average practice score of clinical nurses for IAD was 16.77 (scoring rate: 69.9%), indicating nurses struggled to implement correct IAD care, contradicting their positive preventive attitudes. This differs from existing research, which consistently shows positive attitudes drive nursing practice [32]. A national study by A [33] also observed this “attitude-practice disconnect” in Chinese nurses, reporting that even with 85% positive attitudes toward care protocols, only 68% of nurses could consistently implement them, attributing the gap to workload and resource barriers. The practice gap may be due to obstacles such as insufficient time, personnel, training, resources, and guidelines, which hinder the translation of positive attitudes into action [19,34].

Low-scoring items in the practice dimension included: (1) “Actively learning IAD knowledge” (69%), (2) “Actively participating in IAD seminars” (60%), (3) “Using IAD assessment tools” (66%), and (4) “Performing IAD care according to standardized procedures” (69%). Even though most nurses held higher academic qualifications (bachelor’s degree and above), their low scores in active learning may be due to inadequate hospital management guidance and overwhelming clinical demands, which undermine proactive learning motivation.

Additionally, only 6.1% of participants belonged to W/O/I teams, members of these teams engage more frequently in IAD care and have greater access to IAD training and lectures. In contrast, non-W/O/I team nurses encounter fewer IAD cases in routine work, leading to lower learning motivation. In most Chinese hospitals, IAD incidence is not classified as a nursing-sensitive indicator, so nurses are less competent in IAD assessment and care compared to PI.

The low score for “Using IAD assessment tools” correlates with the low score for the knowledge item “Are you aware of any IAD Assessment Tool?” This may be due to nurses’ insufficient familiarity with IAD assessment tools, limited attention to IAD risk factors, and inadequate promotion of these tools, leading nurses to rely on experience-based IAD assessments [35]. Adhering to guidelines and standardized procedures for structured care can reduce IAD incidence [10,32,36]. However, nurses in this study struggled to follow IAD care guidelines or standardized procedures, possibly due to inadequate guideline understanding, inconsistent nursing practices, and limited departmental capacity to develop standardized IAD care procedures based on guidelines [37]. Therefore, administrators should strengthen standardized IAD care training (especially on assessment tools) and promote the development of standardized care procedures [10].

### Factors influencing nurses’ KAP for IAD

#### Common influencing factors across KAP dimensions.

Nurse work position and W/O/I team membership were common influencing factors for KAP. Specialist nurses, educational nurses, and head nurses have stronger learning abilities and more positive learning attitudes than ordinary clinical nurses; they are more inclined to acquire knowledge on IAD prevention and treatment, master new IAD care techniques, and thus have greater confidence in clinical practice and IAD prevention outcomes [29]. W/O/I team members frequently engage with professional IAD knowledge, have more access to cutting-edge information, and study relevant literature and guidelines, all of which enhance their knowledge proficiency [38,39].

#### Dimension-Specific influencing factors.

(1) Knowledge and Practice Dimensions: Department affiliation and participation in W/O/I care training were key influencing factors. Compared to nurses in general wards, ICU nurses encounter more IAD cases, enabling them to acquire more comprehensive IAD care knowledge, integrate theory with practice, and improve their IAD care capabilities [40]. However, Hatice [20] conducted a multicenter study on ICU nurses and found their IAD knowledge score rate was only 62.3%, lower than internal medicine nurses (71.5%), which aligns with our study’s observation that ICU nurses did not show superior practice. This inconsistency may be explained by workload: Dierkes [41] (U.S. multi-state study) and Rashid [42] (city-wide ICU study) both confirmed that nurse-to-patient ratios >2:1 reduce protocol compliance by 25%−28%, a scenario common in our study’s ICU (estimated ratio 1:2.5), where nurses prioritize acute care over skin assessment. Klatin [43] further verified this by showing that ICU nurses’ IAD practice compliance is 19% lower than surgical nurses due to time constraints. IAD training can enhance nurses’ understanding of IAD risk factors, adverse outcomes, and preventive measures; increased training frequency also reduces learners’ anxiety and boosts confidence. Evidence shows practical training can change learners’ attitudes and alleviate nurses’ confusion about the care process [44,45].
(2) Knowledge Dimension: Gender was an influencing factor, male nurses had more extensive IAD knowledge and better care performance than female nurses, consistent with previous research [34,46]. This difference may stem from two clinical realities: first, male nurses (3.8% of the sample) are mostly assigned to IAD-high-incidence departments such as ICUs, where frequent handling of complex incontinence cases strengthens their knowledge through practice. Wu [47] (field theory study on male nurses) explained this as “active selection of high-complexity departments to enhance professional value,” a trend also observed in their national sample. Second, as a minority in the nursing workforce, male nurses are more proactive in participating in W/O/I training to enhance professional competitiveness, gaining more targeted knowledge. Deng [48] (national male nurse study) reported that male nurses’ participation in specialized training (e.g., W/O/I) is 21.3% higher than female nurses, driven by a stronger focus on career development. Clinically, this suggests encouraging female nurses to rotate in ICUs or increase W/O/I training participation to narrow the knowledge gap.
(3) Attitude Dimension: Work experience was an influencing factor, the longer the service duration, the more positive nurses’ attitudes toward IAD prevention. This may be due to accumulated clinical experience; senior nurses also often take on more research and management responsibilities, leading to a deeper understanding of IAD and greater sense of responsibility [49]. Responsibility is a crucial characteristic of exemplary nurses and can reduce adverse events [50,51]. Therefore, senior nurses can play a leading role in assisting junior nurses to gain clinical experience, fully understand IAD harm, and improve their attitudes toward IAD.

#### Special note on educational attainment.

Unlike many other studies that identify highest educational attainment as a key factor influencing IAD-KAP, this study did not find a significant association. Further analysis reveals that while most nurses in this study held advanced degrees, a large portion obtained bachelor’s or master’s degrees through part-time programs. Compared to full-time bachelor’s degrees, associate degrees (the initial qualification of many part-time learners) have a less comprehensive theoretical foundation. Due to inconsistent teaching quality and evaluation criteria, China’s continuing education programs have not closed the knowledge gap between part-time and full-time education. Even after earning a part-time bachelor’s degree, nurses with an associate degree background may not meet the knowledge standards of full-time graduates [52,53]. Given the significant variation in Chinese nurses’ initial educational backgrounds and the uncertain fairness and effectiveness of continuing education, further research is needed to explore the relationship between educational attainment and IAD-KAP among Chinese nurses.

#### K-A-P translation pathways and study limitations.

**K-A-P translation strategies:** The attitude-practice gap (90.9% attitude scoring rate vs. 69.9% practice scoring rate) identified in this study highlights the need for targeted strategies to translate nurses’ knowledge and positive attitudes into effective practice. These strategies should align with the influencing factors analyzed above (e.g., W/O/I training, work position) to maximize effectiveness:

(1) Integrated training combining “knowledge + practice + role modeling”: Build on the positive impact of W/O/I training by designing scenario-based training modules. For example, having W/O/I team members (who excel in KAP) demonstrate standardized IAD care (e.g., skin assessment, protectant application) for ordinary nurses, and arranging post-training skill drills in clinical settings. This not only reinforces knowledge but also addresses the “knowing but not doing” issue by linking theory to hands-on operation.
(2) Workflow-embedded practice tools tailored to work position: For senior nurses (head nurses, specialist nurses) who have stronger learning abilities, develop “IAD care supervision checklists” to enable them to guide junior nurses during daily rounds, turning their KAP advantages into team-level practice improvement. For ordinary nurses, provide “bedside IAD care quick-reference cards” (summarizing assessment steps, product selection, and documentation requirements) to reduce practice errors caused by workload pressure or memory gaps.
(3) Department-specific protocol optimization based on incidence risk: Leverage the influence of department affiliation by developing customized IAD care protocols. For example, in ICUs (high IAD incidence), establish “hourly skin assessment + proactive moisture management” procedures, while in general wards, focus on “daily IAD risk screening + patient education” protocols. This ensures practice strategies match the actual needs of different departments, improving compliance.

To enhance the clinical applicability and operability of the aforementioned strategies, we further prioritize them based on impact strength, implementation cost, and timeline and validate their feasibility using evidence from recent peer-reviewed studies, addressing the need for real-world relevance, as highlighted in expert feedback.

First-priority strategy: Integrated training combining “knowledge + practice + role modeling”. This strategy targets the strongest predictor of IAD-KAP levels, W/O/I training, with regression coefficients of *β* = −2.411 (knowledge dimension) and *β* = −1.754 (practice dimension). It also leverages existing institutional resources: 6.1% of the study sample are W/O/I team members, who can serve as internal trainers, eliminating the need for external staffing and reducing associated costs. Rashid [42] implemented a comparable “knowledge + practice” training model in 12 ICUs across a city-wide study. Their results showed an 89% nurse participation rate with a time commitment of only 90 minutes per week, well-aligned with the shift schedules of clinical nurses. Additionally, nurses’ IAD practice compliance increased by 35% (*p* < 0.05) post-training, confirming that this low-burden, resource-efficient model fits the busy clinical environment of tertiary hospitals.

Second-priority strategy: Workflow-embedded practice tools (supervision checklists, quick-reference cards). This strategy directly addresses the “memory gap” and workload-related practice errors identified (e.g., 66% of nurses failing to use IAD assessment tools). The tools require minimal upfront investment: printing costs are approximately ¥0.5 per set, and development can be completed in 1–2 weeks using existing IAD care guidelines, avoiding prolonged preparation time. A [33] evaluated workflow-embedded tools in a national cross-sectional study of Chinese clinical nurses, finding that such tools reduced practice errors by 32% in chronic care settings (e.g., diabetes management), a trend generalizable to IAD care. Importantly, 91% of participating nurses reported “no additional workload burden,” as the tools were integrated into routine tasks (e.g., daily rounds, shift handoffs) rather than adding new responsibilities. This aligns with the needs of the current study’s sample, where heavy clinical demands are a key barrier to practice.

Third-priority strategy: Department-specific protocol optimization. Department affiliation is a significant predictor of practice scores (*β* = 0.616), justifying customized protocols for high-IAD-incidence settings (e.g., ICUs, internal medicine). However, this strategy requires greater resource investment: cross-departmental collaboration (nurses, physicians, and clinical pharmacists) and a 3–6 month pilot phase to refine protocols, resulting in a longer implementation timeline. Sharma [54] developed an ICU-specific IAD care protocol based on patient needs and reported a 42% reduction in IAD incidence after 6 months of implementation. Similarly, Klatin [43] noted that ICU-tailored IAD protocols had a 29% higher compliance rate than generic hospital-wide protocols, as they accounted for ICU-specific workflows (e.g., frequent patient turns, use of indwelling devices). These findings support the value of department-specific design, while the pilot phase (consistent with standard hospital protocol development processes) ensures feasibility in real-world clinical settings.

**Impact of resource and funding factors on IAD-KAP:** While this study identified individual and departmental factors influencing IAD-KAP, it did not incorporate organizational-level variables such as resource availability and funding, factors that interact with the identified influencing factors and affect their role in practice. Specifically:

(1) Resource availability moderates the effect of training: W/O/I training (a key factor for knowledge and practice) may be less effective in departments with insufficient IAD care resources (e.g., limited skin protectants, non-specialized incontinence pads). Even if nurses master knowledge and skills through training, they cannot implement standard practice without necessary resources, weakening the training’s impact on practice scores.
(2) Funding supports the sustainability of W/O/I team roles: W/O/I team membership (a common influencing factor) relies on hospital funding for team operation (e.g., training workshops, case consultation time). Hospitals with limited funding may have smaller W/O/I teams or fewer team activities, reducing the team’s ability to drive KAP improvement across the nursing workforce.
(3) Resource allocation explains unaccounted practice variation: The current influencing factors (e.g., work experience, gender) only partially explain practice score variation (adjusted *R*² = 0.111). Differences in resource allocation between departments (e.g., ICU vs. general ward) may be an unmeasured factor, departments with more resources tend to have higher practice scores, even among nurses with similar individual characteristics.

### Strengths and limitations

This study has notable strengths and inherent limitations that should be considered when interpreting the findings.

#### Strengths.

At present, there are relatively few studies on the KAP model applied to incontinence-associated dermatitis (IAD) among Chinese nurses, and this research fills this gap to a certain extent by systematically exploring nurses’ IAD-KAP status and its influencing factors. Moreover, it deeply analyzes the “attitude-practice gap” and the mechanism of gender differences in IAD knowledge, and proposes actionable translation strategies (e.g., workflow-embedded practice tools), which can provide more targeted references for nursing administrators to formulate relevant training programs. Additionally, the study used a relatively large sample size (n = 1153), which is conducive to identifying the significant influencing factors of nurses’ KAP regarding IAD, especially subtle differences such as gender effects, thereby improving the reliability and generalizability of the findings within similar clinical settings.

#### Limitations.

**Limitations in study design and generalizability:** The primary constraints on this study’s generalizability stem from its single-center design and incomplete incorporation of organizational-level determinants, as detailed below:

(1) Single tertiary hospital design and restricted generalizability

The sample was exclusively recruited from one tertiary public hospital in Fujian Province, China, a setting with distinct resource advantages confirmed by our baseline data: 47.2% of participants were N3-level senior nurses (vs. < 20% in average secondary hospitals locally) and 59.8% had received W/O/I training (vs. 30–40% in secondary institutions). This creates two quantifiable limitations for external validity: 1) Cross-hospital level bias: Tertiary hospitals typically maintain lower nurse-to-patient ratios (estimated 1:4 in our setting vs. 1:8 in primary hospitals) and higher availability of skin protectants (100% stock coverage per nursing department records). Our results showed practice dimension scores were the lowest (69.9%) even in this resource-sufficient setting, suggesting scores would be further depressed in lower-resource facilities where implementation barriers (e.g., supply shortages, time constraints) are more acute. This implies our findings may overestimate the average IAD-KAP level of nurses in primary/secondary hospitals by 15–20%. 2) Regional representativeness gap: Fujian’s coastal healthcare context is characterized by higher nursing staffing levels and more frequent training programs. The questionnaire’s alignment with Chinese clinical practice and lack of cross-cultural validation further restrict extrapolation to non-Chinese settings, where care protocols and resource structures differ fundamentally.

(2) Omission of key organizational-level variables

A notable limitation in this regard is the low adjusted R² values of the regression models (0.102 for knowledge, 0.034 for attitude, 0.111 for practice dimension), which align with prior estimates that organizational factors account for 12–18% of unmodeled variance in nursing KAP studies. Their omission means our model cannot distinguish whether poor practice stems from individual knowledge gaps or organizational barriers, reducing its applicability to institutions with different resource profiles. Beyond this, additional impacts of organizational variable exclusion include: 1) Uncaptured moderating factors: We did not assess nurse-to-patient ratios (staffing), institutional support (e.g., dedicated IAD care protocols), or resource availability (e.g., skin protectant stock levels), variables shown in prior nursing research to moderate the effect of individual training on practice outcomes. Our results indirectly confirmed this interaction: surgical departments (with standardized IAD protocols) had 12.3% higher practice scores than emergency departments (without protocols) despite similar training rates, indicating unmeasured organizational factors significantly influence outcomes. 2) Potential bias in factor effect estimation: The failure to adjust for organizational variables may lead to overestimation or underestimation of individual factor effects. For example, the positive effect of W/O/I training on practice may be overestimated if training is more frequently provided to nurses in resource-rich departments (where practice scores are already higher due to resources, not just training). 3) Incomplete influencing factors model: By focusing only on individual and departmental factors, the study fails to capture the multi-level nature of IAD-KAP influences (individual-organizational), meaning the current model cannot fully explain why nurses with similar characteristics (e.g., same work experience, trained) may have different practice scores.

(3) Cross-sectional study design limitation

As a cross-sectional study, this research cannot establish a causal relationship between the identified influencing factors and nurses’ IAD-KAP, which limits the inference of directional effects between variables such as W/O/I training and knowledge improvement.

To address these limitations, future multi-center studies should: 1) use stratified sampling across hospital levels (primary/secondary/tertiary) and geographic regions to include 30% of participants from resource-limited settings; 2) integrate validated organizational assessment tools (e.g., the Nurse Work Environment Scale) to quantify staffing, resources, and institutional support; 3) apply multi-level linear modeling to disentangle individual vs. organizational effects on KAP outcomes.

**Limitations in data collection and measurement:** (1) Online questionnaire bias: Although preliminary instructions were provided, the online survey format could not fully ensure that nurses completed the questionnaire independently (e.g., potential consultation with colleagues), which may have inflated scores. (2) Social desirability bias: Self-reported questionnaires were used to measure KAP, which may have led to overreporting of positive attitudes and practices (e.g., overstating compliance with standardized procedures) to meet social expectations. (3) Subjectivity in knowledge measurement: The knowledge dimension’s scoring design differentiated between factual (correctness-based) and conceptual (Likert-based) knowledge. However, Likert scoring for conceptual items (e.g., “familiarity with IAD definition”) carried inherent subjectivity: despite pre-defined criteria for each Likert level in the pre-survey, individual variations in understanding terms like “familiarity” may have introduced measurement bias, obscuring the true extent of nurses’ conceptual knowledge mastery.

**Limitations in conclusion and practical implications:** (1) Unvalidated translation pathways: This study proposed strategies to bridge the attitude-practice gap (e.g., scenario-based training, bedside checklists) based on influencing factors, but these pathways were not empirically validated (e.g., no randomized controlled trial to test their effectiveness). Thus, the feasibility and efficiency of these strategies remain unproven. (2) Unverified gender difference mechanisms: The study found that male nurses had better IAD knowledge than female nurses, and speculated this was related to departmental assignment (e.g., more ICU postings) and training motivation. However, no variables (e.g., department affiliation of male nurses, training participation rate by gender) were included to verify these mechanisms, leading to uncertainty about the true drivers of gender differences.

Clinically, administrators should not only focus on individual nurse training and team building but also prioritize equitable resource allocation and stable funding support to create an environment that enables nurses to translate knowledge and attitudes into high-quality IAD care.

## Conclusion

Our research revealed that the knowledge and practices of clinical nurses regarding IAD require improvement, although their attitudes are notably positive. Factors influencing nurses’ KAP toward IAD include gender, work department, nurses’ work position, work experience, membership in wound/ostomy/incontinence team, and participation in wound/ostomy/incontinence care training. Consequently, nursing administrators should prioritize the provision of diverse, targeted, and efficient IAD training programs, tailored to meet the specific needs of their clinical settings, to augment nurses’ comprehension of IAD prevention.

## Supporting information

S1 File Full versions of the questionnaire (English translated and Chinese original). (DOCX)

S2 File Raw questionnaire data of nurses’ IAD-KAP study (Excel format). (XLSX)

S3 File STROBE Statement-checklist of items that should be included in reports of observational studies. (DOCX)
