# Supplementary material for: Clinical nurses’ knowledge, attitudes and practices regarding incontinence-associated dermatitis: A cross-sectional study from China
Source: PLoS One. 2025 Dec 9;20(12):e0337721. doi: 10.1371/journal.pone.0337721 (PMC12688137; doi:10.1371/journal.pone.0337721)
Supplement: S1 File — (DOCX) [file pone.0337721.s001.docx]

**S1-1 File. The full English translated version of the questionnaire.**

Dear nursing colleagues,

Hello! The purpose of this survey is to understand your knowledge, attitude and behavior of incontinence dermatitis, and to provide reference for subsequent specialist training and clinical application. This survey will strictly abide by the principles of informed consent, anonymity and confidentiality. It is only for the status quo investigation, and the survey results will not be used for other purposes. Please fill in the survey truthfully.

Fill in instructions: 1. Please tick the corresponding box in accordance with your wishes. 2. For other questions, please fill in the corresponding content directly.

General information questionnaire

1. Gender: □ female □ male

2. Age (years) : □ 20-30 □31-40□41-50□≥51□

3. Job grade : □ N0 □ N1 □ N2 □ N3 □ N4

4. Nurses work position: □ Staff nurse (clinical) □ Education □ Head nurse (management) □ Wound care nurse

5. Highest level of education: □ Associate degree□ Undergraduate □ Master and above

6. Department: □ Department of surgery□ Department of internal medicine □ Emergency room □ ICU

7. Work experiences (year) : □ < 5 □6-10 □11-15 □16-20 □≥20

8. Are you a member of a wound/ostomy/incontinence team? : □ Yes □ no

9. Have you attended any wound/ostomy/incontinence care training? : □ None □ yes

1. **Knowledge related to incontinence dermatitis**

1. How much do you know about the definition of incontinence dermatitis?

A knows B better than C and D doesn't

2. Which of the following factors do you think is related to the occurrence of incontinence dermatitis?

A Incontinence B friction C pH environment D age

3. Which of the following body fluids do you think is the most damaging to the skin

A urine B formed stool C watery stool D sweat

4. Clinical manifestations of regional skin with incontinence dermatitis

A Erythema with unclear borders B pruritus pain C lesions D ulcers with clear edges

5, Do you know the difference between incontinence dermatitis and pressure sore?

A knows B better than C and D doesn't

6. Do you know the classification of incontinence dermatitis

A knows B better than C and D doesn't

7. Do you know the incontinence dermatitis assessment tool

A knows B better than C and D doesn't

8. What do you think about the prevention and care of incontinence dermatitis

A Remove skin irritants B Use devices or products to remove skin from moisture

C To prevent secondary skin infections D to control or divert the causes of skin dampness

9. What steps do you think are more important to prevent incontinence

A Clean B moisturize C protect D are all above

10, do you think that incontinence dermatitis skin cleaning equipment which is more appropriate

A daily use basin and towel B- secondary basin and towel C Clean basin and towel D casually

11. What kind of skin cleaning solution do you think is more suitable for incontinence dermatitis

A Soap and water B Clean water C Special leave-in detergent D saline solution

12. Which products do you use most often in the care of incontinence dermatitis

A skin protective film B powder C oil paste D liquid

13. What incontinence care products have you used

A indurating catheter or diaper B diaper or pad C anal adhesive ostomy bag D Self-made catheter device inserted into the anus

**(2) Your attitude towards clinical incontinence dermatitis:**

1. You think it is very important for the hospital to train the knowledge of incontinence dermatitis

A strongly agrees with B, more agree with C, generally agree with D disagree

2. You think it is very important to participate in training to improve the prevention and treatment of incontinence dermatitis by nurses

A strongly agrees with B, more agree with C, generally agree with D disagree

3. You think prevention of incontinence dermatitis is more important than treatment

A strongly agrees with B, more agree with C, generally agree with D disagree

4. You think the choice of incontinence care tools is very important in the prevention and treatment of incontinence dermatitis

A strongly agrees with B, more agree with C, generally agree with D disagree

5. You think that skin protection measures are very important in the prevention and treatment of incontinence dermatitis

A strongly agrees with B, more agree with C, generally agree with D disagree

6. You think it is very important to strengthen the care capacity of family members/caregivers for the prevention and treatment of incontinence dermatitis

A strongly agrees with B, more agree with C, generally agree with D disagree

7. You think it is very important to develop standardized care procedures for incontinence dermatitis

A strongly agrees with B, more agree with C, generally agree with D disagree

**(3)Your behavior in incontinence dermatitis:**

1, whether you have taken the initiative to learn the relevant knowledge of incontinence dermatitis

A often B sometimes C rarely D never

2. Have you taken the initiative to participate in academic lectures or training on incontinence dermatitis

A often B sometimes C rarely D never

3, whether you targeted family members/caregivers to incontinence dermatitis related knowledge education

A often B sometimes C rarely D never

4. Do you care for patients with incontinence dermatitis according to standardized procedures

A often B sometimes C rarely D never

5. Have you used an incontinence care assessment tool

A often B sometimes C rarely D never

6, whether you have used skin protection tools

A often B sometimes C rarely D never

**S1-2 File. The full Chinese original version of the questionnaire.**

尊敬的各位护理同仁：

您好！本调查是为了解您对失禁性皮炎相关知识、态度、行为的认知程度，为后续专科培训 及临床应用工作的提供参考依据。此次调查将严格遵守知情同意、匿名、保密的原则，仅为 现状调查，调查结果不作他用，请如实填写，希望等到您的帮助，感谢您的配合！

填写说明：１.请您在符合意愿的相应方格内打 √。２.其他题型请您直接填写相应内容。

一般资料调查问卷

1.性别： □女 □男

2.年龄（岁）： □ 20-30 □31-40□41-50□≥51□

3.职称：□ N0 □ N1 □ N2 □ N3 □ N4

4.职务：□ 临床护士 □ 教育护士 □ 护士长 □ 伤口专科护士

5.学历：□大专 □本科 □硕士及以上

6.科室：□外科 □内科 □急诊 □ICU

7.工作年限（年）：□ < 5 □6-10 □11-15 □16-20 □≥20

8.你是否为伤口/造口/失禁小组成员：□是 □否

9.参加伤口培训： □无 □有

一、失禁性皮炎相关知识

1、您对失禁性皮炎定义的了解程度？

Ａ 熟知 Ｂ 比较了解 Ｃ 知道一点 Ｄ不知道

1. 您认为失禁性皮炎的发生与下列哪些因素有关？

Ａ 失禁 Ｂ 摩擦力 Ｃ 酸碱环境 Ｄ年龄

1. 您认为下列哪些体液对皮肤损伤最大

Ａ 尿液 Ｂ成形的粪便 Ｃ水样的粪便 Ｄ汗液

1. 失禁性皮炎区域皮肤临床表现

Ａ红斑且边界不清 Ｂ瘙痒疼痛 Ｃ皮损 Ｄ边缘清晰的溃疡

1. 您是否知道失禁性皮炎与压疮的鉴别？

Ａ熟知 Ｂ比较了解 Ｃ知道一点 Ｄ不知道

1. 您是否知道失禁性皮炎的分级

Ａ熟知 Ｂ比较了解 Ｃ知道一点 Ｄ不知道

1. 您是否知道失禁性皮炎评估工具

Ａ熟知 Ｂ比较了解 Ｃ知道一点 Ｄ不知道

1. 您认为失禁性皮炎的预防与护理要点

Ａ 移除皮肤刺激物 Ｂ 使用器械或产品让皮肤脱离潮湿环境

Ｃ 预防继发性皮肤感染 Ｄ 控制或转移引起皮肤潮湿的原因

1. 您认为预防失禁性皮下面哪些步骤较为重要

Ａ 清洗 Ｂ 滋润 Ｃ 保护 Ｄ 以上都是

1. 您认为失禁性皮炎清洗皮肤的用具哪种较为合适

Ａ日常用盆及毛巾 Ｂ－次性盆及毛巾 Ｃ清洗干净的盆及毛巾 Ｄ随便

1. 您认为失禁性皮炎的皮肤清洁溶液哪种较为合适

Ａ肥皂加水 Ｂ 清水 Ｃ 专用免洗清洁剂 Ｄ生理盐水

1. 您在护理失禁性皮炎中最常使用哪些产品

Ａ 皮肤保护膜 Ｂ 粉剂 Ｃ油 膏类 Ｄ液体类

1. 您在失禁护理用品方面使用过哪些

Ａ 留置导尿或尿套 Ｂ纸尿裤或尿垫 C肛门黏贴造口袋 Ｄ自制导管装置插入肛门

1. 您对临床失禁性皮炎的态度：
2. 您认为医院进行失禁性皮炎知识培训非常重要

Ａ 非常同意 Ｂ比较同意 Ｃ一般同意 Ｄ不同意

1. 您认为参加培训对于提高护士防治失禁性皮炎非常重要

Ａ 非常同意 Ｂ比较同意 Ｃ一般同意 Ｄ不同意

1. 您认为失禁性皮炎预防比治疗更重要

Ａ 非常同意 Ｂ比较同意 Ｃ一般同意 Ｄ不同意

1. 您认为失禁护理工具选择在失禁性皮炎防治方面非常重要

Ａ 非常同意 Ｂ比较同意 Ｃ一般同意 Ｄ不同意

1. 您认为皮肤保护措施在失禁性皮炎防治方面非常重要

Ａ 非常同意 Ｂ比较同意 Ｃ一般同意 Ｄ不同意

1. 您认为加强家属／护工的照护能力对失禁性皮炎防治非常重要

Ａ 非常同意 Ｂ比较同意 Ｃ一般同意 Ｄ不同意

1. 您认为制定标准化的失禁性皮炎护理流程非常重要

Ａ 非常同意 Ｂ比较同意 Ｃ一般同意 Ｄ不同意

三、您在失禁性皮炎上的相关行为：

1、您是否主动去学习过失禁性皮炎的相关知识

Ａ 经常 Ｂ 有时 Ｃ很少 Ｄ从不

1. 您是否主动参加过有关失禁性皮炎学术讲座或培训

Ａ 经常 Ｂ 有时 Ｃ很少 Ｄ从不

1. 您是否针对性地给家属／护工进行失禁性皮炎相关知识的宣教

Ａ 经常 Ｂ 有时 Ｃ很少 Ｄ从不

4、您是否根据标准化的流程对失禁性皮炎病人进行护理

Ａ 经常 Ｂ 有时 Ｃ很少 Ｄ从不

5、您是否使用过失禁护理评估工具

Ａ 经常 Ｂ 有时 Ｃ很少 Ｄ从不

6、您是否使用过皮肤保护工具

Ａ 经常 Ｂ 有时 Ｃ很少 Ｄ从不
