# Supplementary material for: Clinical nurses’ knowledge, attitudes and practices regarding incontinence-associated dermatitis: A cross-sectional study from China
Source: PLoS One. 2025 Dec 9;20(12):e0337721. doi: 10.1371/journal.pone.0337721 (PMC12688137; doi:10.1371/journal.pone.0337721)
Supplement: S3 File — (DOCX) [file pone.0337721.s003.docx]

S3 File. STROBE Statement—checklist of items that should be included in reports of observational studies

|  | Item No. | Recommendation | Page  No. | Relevant text from manuscript |
| --- | --- | --- | --- | --- |
| **Title and abstract** | 1 | (*a*) Indicate the study’s design with a commonly used term in the title or the abstract | 1 | Cross-sectional study. |
|  |  | (*b*) Provide in the abstract an informative and balanced summary of what was done and what was found | 1-2 | The purpose of this article is to explore the current status of clinical nurses' knowledge, attitudes, and practices regarding incontinence-associated dermatitis in China, and to analyze the factors that influence them. |
| Introduction | | | |  |
| Background/rationale | 2 | Explain the scientific background and rationale for the investigation being reported | 3-5 | Incontinence-associated dermatitis (IAD) is an irritant dermatitis of the gluteal, sacrum-caudal, and perianal regions resulting from prolonged exposure to urine and feces[1]. IAD is characterized by diffuse erythema, usually with or without edema of the skin surface[2,3]. As one of the most prevalent forms of moisture-associated dermatitis, IAD has evolved into a global health concern, necessitating increased attention from healthcare professionals to focus on prevention and mitigation strategies |
| Objectives | 3 | State specific objectives, including any prespecified hypotheses | 5 | Given the high incidence of IAD in China and its adverse effects on patients, this study aims to systematically explore the IAD-related KAP of Chinese clinical nurses. The findings will provide evidence-based data for nursing administrators, supporting the development of targeted training programs. Ultimately, this will enhance Chinese nurses' KAP of IAD and improve the quality of IAD prevention in clinical settings. |
| Methods | | | |  |
| Study design | 4 | Present key elements of study design early in the paper | 5 | This study explored nurses’ knowledge, attitude, and practice in IAD. A cross‐sectional design using an anonymous online survey was conducted. The findings were reported using the STROBE checklist. |
| Setting | 5 | Describe the setting, locations, and relevant dates, including periods of recruitment, exposure, follow-up, and data collection | 5 | This study explored nurses’ knowledge, attitude, and practice in IAD. A cross‐sectional design using an anonymous online survey was conducted. The findings were reported using the STROBE checklist. |
| Participants | 6 | (*a*) *Cohort study*—Give the eligibility criteria, and the sources and methods of selection of participants. Describe methods of follow-up  *Case-control study*—Give the eligibility criteria, and the sources and methods of case ascertainment and control selection. Give the rationale for the choice of cases and controls  *Cross-sectional study*—Give the eligibility criteria, and the sources and methods of selection of participants | 5-6 | The whole nursing staff of a tertiary hospital in Fujian, China, comprises the statistical population for this study. This Chinese tertiary hospital is a general hospital with more than 2000 beds that offers specialized medical care and functions as a regional medical center. The practical sample approach was used to choose the clinical nurses who offered to take part in this study at the hospital. The inclusion and exclusion criteria for participants were as follows: Inclusion criteria included: (1) being a registered nurse; (2) having at least six months of clinical work experience; (3) volunteering to participate in the study. Exclusion criteria included: (1) nurses from other hospitals who come to our hospital for further education; (2) providing insufficient information in the questionnaire, including either missing key data on essential study variables (e.g., job position, membership in the wound/ostomy/incontinence team) or incomplete responses to more than 15% of the total questionnaire items, which would compromise the reliability of the K-A-P assessment. |
|  |  | (*b*) *Cohort study*—For matched studies, give matching criteria and number of exposed and unexposed  *Case-control study*—For matched studies, give matching criteria and the number of controls per case | / | / |
| Variables | 7 | Clearly define all outcomes, exposures, predictors, potential confounders, and effect modifiers. Give diagnostic criteria, if applicable | 6 | A self-report questionnaire was used to gather data on the general (gender, age, education level, work experience, position, job grade, and department) and work-related (attendance at wound, ostomy, and incontinence training courses, and membership in the wound/ostomy/incontinence team) characteristics of the participants. |
| Data sources/ measurement | 8* | For each variable of interest, give sources of data and details of methods of assessment (measurement). Describe comparability of assessment methods if there is more than one group | 6-9 | Utilizing the IAD Knowledge, Attitude, and Practice Questionnaire developed by Chinese scholar Kong Jie, this study assesses the KAP levels of clinical nurses regarding IAD[22]. The questionnaire encompasses three dimensions: knowledge, attitude, and practice, comprising a total of 26 items with a scoring range of 26 to 104 points. In the knowledge dimension, nine items are scored with four points for correct answers, while incorrect or partially correct responses result in a deduction of one point per option. The remaining items employ a 4-point Likert scale ranging from "familiar" to "unfamiliar," scored from four to one point respectively. The attitude dimension also utilizes a 4-point Likert scale, from "strongly agree" to "disagree," with scores assigned from one to four points. Similarly, the practice dimension adopts a 4-point Likert scale, extending from "frequently" to "never," with corresponding scores of four to one point. Three dimensions of Cronbach's α yielded values of 0.889, 0.879, and 0.874, with a total Cronbach's α of 0.889, indicating moderate to high test-retest reliability, as the mean CVI fluctuated within the range of 0.9 to 1, while content validity and reliability were evaluated at 0.837 and 0.969, respectively, suggesting satisfactory validity and appropriateness for a KAP survey among clinical nursing personnel. Scoring rate = average score/perfect score *100%, scoring rate >80% is good (positive), scoring rate 60%–80% is medium (neutral), and scoring rate < 60% is poor (negative). |
| Bias | 9 | Describe any efforts to address potential sources of bias | 8-9 | The questionnaire was distributed online between June 12, 2024, and July 12, 2024 (http://ww.wjx.cn). The QR code was distributed through WeChat groups. Before the survey began, the researchers organized the heads of various departments to attend offline training sessions. They introduced the study in detail, including the purpose of the survey, the qualifications of the participants, and precautions. The person in charge of each department is required to organize nurses to complete the questionnaire with high quality. After getting consent forms from the participants, respondents answered the questionnaire anonymously using electronic devices such as laptops and mobile phones. The participants were further informed of this voluntary survey by reading through the introduction section of the survey questionnaire, which added the background of the study, the purpose, data privacy protection, and gratefulness for their participation. When completing the questionnaire, the participants can consult the researcher if they have questions. |
| Study size | 10 | Explain how the study size was arrived at | / | / |

Continued on next page

| Quantitative variables | 11 | Explain how quantitative variables were handled in the analyses. If applicable, describe which groupings were chosen and why | 9-12 | For data analysis, IBM's Statistical Package for Social Science (SPSS; 27.0) was utilized. The study applied descriptive statistics, such as frequency, percentage, mean, and standard deviation, to examine the overall attributes of the participants. Furthermore, t-tests and ANOVA were utilized for univariate analyses for every dimension. In cases where the data did not fit a normal distribution, two-sided Mann-Whitney U tests and Kruskal-Wallis H tests were employed for the studies. The association between knowledge, attitude, and behavior was examined by determining the value of Pearson product-moment correlation coefficients. A multivariate stepwise regression analysis was performed on all variables that had a P-value of less than 0.05 in the univariate analysis to investigate the factors that influence nurses' knowledge, attitude, and behavior scores (the dependent variable). It was considered statistically significant with P < 0.05. |
| --- | --- | --- | --- | --- |
| Statistical methods | 12 | (*a*) Describe all statistical methods, including those used to control for confounding | 9-12 | For data analysis, IBM's Statistical Package for Social Science (SPSS; 27.0) was utilized. The study applied descriptive statistics, such as frequency, percentage, mean, and standard deviation, to examine the overall attributes of the participants. Furthermore, t-tests and ANOVA were utilized for univariate analyses for every dimension. In cases where the data did not fit a normal distribution, two-sided Mann-Whitney U tests and Kruskal-Wallis H tests were employed for the studies. The association between knowledge, attitude, and behavior was examined by determining the value of Pearson product-moment correlation coefficients. A multivariate stepwise regression analysis was performed on all variables that had a P-value of less than 0.05 in the univariate analysis to investigate the factors that influence nurses' knowledge, attitude, and behavior scores (the dependent variable). It was considered statistically significant with P < 0.05. |
|  |  | (*b*) Describe any methods used to examine subgroups and interactions | / | / |
|  |  | (*c*) Explain how missing data were addressed | / | / |
|  |  | (*d*) *Cohort study*—If applicable, explain how loss to follow-up was addressed  *Case-control study*—If applicable, explain how matching of cases and controls was addressed  *Cross-sectional study*—If applicable, describe analytical methods taking account of sampling strategy | 9-12 | For data analysis, IBM's Statistical Package for Social Science (SPSS; 27.0) was utilized. The study applied descriptive statistics, such as frequency, percentage, mean, and standard deviation, to examine the overall attributes of the participants. Furthermore, t-tests and ANOVA were utilized for univariate analyses for every dimension. In cases where the data did not fit a normal distribution, two-sided Mann-Whitney U tests and Kruskal-Wallis H tests were employed for the studies. The association between knowledge, attitude, and behavior was examined by determining the value of Pearson product-moment correlation coefficients. A multivariate stepwise regression analysis was performed on all variables that had a P-value of less than 0.05 in the univariate analysis to investigate the factors that influence nurses' knowledge, attitude, and behavior scores (the dependent variable). It was considered statistically significant with P < 0.05. |
|  |  | (*e*) Describe any sensitivity analyses | / | / |
| Results | | | | |
| Participants | 13* | (a) Report numbers of individuals at each stage of study—eg numbers potentially eligible, examined for eligibility, confirmed eligible, included in the study, completing follow-up, and analysed | 12 | Participants were nurses from a tertiary public hospital in Fujian Province. A total of 1153 nurses participated. |
|  |  | (b) Give reasons for non-participation at each stage | / | / |
|  |  | (c) Consider use of a flow diagram | / | / |
| Descriptive data | 14* | (a) Give characteristics of study participants (eg demographic, clinical, social) and information on exposures and potential confounders | 12-13 | The majority of the nurses were female (1109, 96.2%). Ages predominantly ranged from 20–30 (407, 35.3%) to 31–40 years (526, 45.6%). Most of the nurses in this study had between 6-10 (297, 25.8%) and 11-15 years (305, 26.5%) of work experience. The majority of nurses have a job grade of N3 (544, 47.2%), followed by N2 (208, 18.0%) and N1 (208, 18.0%). About the level of education, most respondents had an undergraduate degree (934, 81.0%), followed by an associate degree (201, 17.4%) and a master's degree (18, 1.6%). Nurses were predominantly in internal medicine (564, 48.9%) and surgery (453, 39.3%), with (1063, 92.2%) in clinical nursing. Only a small proportion of nurses (70, 6.1%) were members of the wound/ostomy/incontinence team, and more than half (690, 59.8%) had attended a wound/ostomy/incontinence care training course. |
|  |  | (b) Indicate number of participants with missing data for each variable of interest | / | / |
|  |  | (c) *Cohort study*—Summarise follow-up time (eg, average and total amount) | / | / |
| Outcome data | 15* | *Cohort study*—Report numbers of outcome events or summary measures over time | */* | */* |
|  |  | *Case-control study—*Report numbers in each exposure category, or summary measures of exposure | */* | */* |
|  |  | *Cross-sectional study—*Report numbers of outcome events or summary measures | 12-15 | Participants were nurses from a tertiary public hospital in Fujian Province. A total of 1153 nurses participated. The majority of the nurses were female (1109, 96.2%). Ages predominantly ranged from 20–30 (407, 35.3%) to 31–40 years (526, 45.6%). Most of the nurses in this study had between 6-10 (297, 25.8%) and 11-15 years (305, 26.5%) of work experience. The majority of nurses have a job grade of N3 (544, 47.2%), followed by N2 (208, 18.0%) and N1 (208, 18.0%). About the level of education, most respondents had an undergraduate degree (934, 81.0%), followed by an associate degree (201, 17.4%) and a master's degree (18, 1.6%). Nurses were predominantly in internal medicine (564, 48.9%) and surgery (453, 39.3%), with (1063, 92.2%) in clinical nursing. Only a small proportion of nurses (70, 6.1%) were members of the wound/ostomy/incontinence team, and more than half (690, 59.8%) had attended a wound/ostomy/incontinence care training course. Table 1 summarizes the demographic and professional characteristics of the participants. |
| Main results | 16 | (*a*) Give unadjusted estimates and, if applicable, confounder-adjusted estimates and their precision (eg, 95% confidence interval). Make clear which confounders were adjusted for and why they were included | 15-22 | This study analyzed variations in nurses’ IAD-related KAP levels using their demographic and professional characteristics as independent variables, with univariate analysis results summarized in Table 3. To control the risk of Type I error in multiple comparisons, the Bonferroni correction was applied for pairwise comparisons among multi-group variables (e.g., age, work experience, job grade), with the corrected significance level set to α' = 0.05/n (where n denotes the number of pairwise comparisons); for two-group variables (e.g., gender, membership in the W/O/I team), two-tailed tests were used to strictly control the significance level.  Pearson correlation analysis was conducted to explore the associations between nurses’ IAD knowledge, attitudes, and practice, with results summarized in Table 4—these relationships provide critical insights for prioritizing IAD care improvement strategies.  To identify key factors affecting nurses’ IAD-KAP levels, multiple linear stepwise regression analyses were conducted, with KAP dimension scores (knowledge, attitude, practice) and total KAP score as dependent variables. Only variables with p < 0.05 in univariate analyses were included as independent variables, and their coding is detailed in Table 5; raw KAP scores were used for regression inputs to preserve result interpretability. To minimize Type I error in multivariable testing, variables were entered into the model at p < 0.05 and removed at p > 0.10. Multicollinearity was assessed using the VIF, with all included variables having VIF < 5—confirming no severe multicollinearity that could distort results. Before interpreting regression outputs, core linear regression assumptions (residual normality and homoscedasticity) were validated via residual-based analyses, as detailed below |
|  |  | (*b*) Report category boundaries when continuous variables were categorized | / | / |
|  |  | (*c*) If relevant, consider translating estimates of relative risk into absolute risk for a meaningful time period | / | / |

Continued on next page

| Other analyses | 17 | Report other analyses done—eg analyses of subgroups and interactions, and sensitivity analyses | / | / |
| --- | --- | --- | --- | --- |
| Discussion | | | | |
| Key results | 18 | Summarise key results with reference to study objectives | 22-27 | Clinical nurses had poor knowledge of the IAD and inadequate nursing behaviors, but still maintained a good attitude. |
| Limitations | 19 | Discuss limitations of the study, taking into account sources of potential bias or imprecision. Discuss both direction and magnitude of any potential bias | 27-30 | This study has several limitations. Our sample was drawn from a tertiary hospital in Fujian Province, China, and the KAP survey questionnaire used in this research was developed based on the Chinese context, lacking validation across different cultures, which may restrict the generalizability of the findings. The study employed an online questionnaire format, despite providing preliminary instructions, it was not entirely possible to ensure that nurses independently completed the survey as required. Lastly, our research may be subject to desirability bias, as we utilized self-reported questionnaires. |
| Interpretation | 20 | Give a cautious overall interpretation of results considering objectives, limitations, multiplicity of analyses, results from similar studies, and other relevant evidence | 22-27 | / |
| Generalisability | 21 | Discuss the generalisability (external validity) of the study results | / | / |
| Other information | |  | | |
| Funding | 22 | Give the source of funding and the role of the funders for the present study and, if applicable, for the original study on which the present article is based | / | / |

*Give information separately for cases and controls in case-control studies and, if applicable, for exposed and unexposed groups in cohort and cross-sectional studies.

**Note:** An Explanation and Elaboration article discusses each checklist item and gives methodological background and published examples of transparent reporting. The STROBE checklist is best used in conjunction with this article (freely available on the Web sites of PLoS Medicine at http://www.plosmedicine.org/, Annals of Internal Medicine at http://www.annals.org/, and Epidemiology at http://www.epidem.com/). Information on the STROBE Initiative is available at www.strobe-statement.org.
